# Type 2 lymphocytes restrict type 3 lymphocytes during liver fibrosis and colocalize in fibroblast niches

**DOI:** 10.1126/sciadv.aea6805

**Published:** 2026-03-11

**Authors:** Julia Sbierski-Kind, Kelly M. Cautivo, Julia Nilsson, Johanna C. Wagner, Madelene W. Dahlgren, Nathan Ewing Crystal, Maria McClave, Nicholas M. Mroz, Marlene Ganslmeier, Carlos O. Lizama, Anna Lu Gan, Peri R. Matatia, Marcela T. Taruselli, Anthony A. Chang, Sofia Caryotakis, Claire E. O’Leary, Maya Kotas, Jun-Hoe Lee, Taeeun Gu, Hyeewon Seo, Hyun Je Kim, Aras N. Mattis, Tien Peng, Richard M. Locksley, Ari B. Molofsky

**Affiliations:** ^1^ Department of Laboratory Medicine, University of California San Francisco, San Francisco, CA 94143, USA.; ^2^ Internal Medicine IV, Department of Diabetology, Endocrinology and Nephrology, University of Tübingen, Otfried-Müller-Str. 10, 72076 Tübingen, Germany.; ^3^ Medical Faculty, The M3 Research Center, University Clinic Tübingen (UKT), Otfried-Müllerstr. 37, 72076 Tübingen, Germany.; ^4^ Institute for Diabetes Research and Metabolic Diseases (IDM) of Helmholtz Munich at the University of Tübingen, Otfried-Müller-Str. 10, 72076 Tübingen, Germany.; ^5^ Department of Surgery, Division of Transplantation, University of California San Francisco, San Francisco, CA 94143, USA.; ^6^ Department of General Visceral, Transplantation, Vascular and Pediatric Surgery, University Hospital Würzburg, Würzburg, Germany.; ^7^ Division of Molecular Hematology, Department of Laboratory Medicine, Lund University, Lund, Sweden.; ^8^ Department of Medicine, Pulmonary Division, University of California San Francisco, San Francisco, CA 94143, USA.; ^9^ Molecular Biology and Microbiology Department, Tufts University School of Medicine, Boston, MA 02111, USA.; ^10^ Graduate Program in Molecular Microbiology, Tufts University Graduate School of Biomedical Sciences, Boston, MA 02111, USA.; ^11^ Biomedical Sciences Graduate Program, University of California San Francisco, San Francisco, CA 94143, USA.; ^12^ Cardiovascular Research Institute, University of California San Francisco, San Francisco, CA 94143, USA.; ^13^ Charité Universitätsklinikum Berlin, Berlin, Germany.; ^14^ Department of Medicine, University of California San Francisco, San Francisco, CA 94143, USA.; ^15^ Department of Pediatrics, School of Medicine and Public Health, University of Wisconsin-Madison, Madison, WI 53792, USA.; ^16^ Quantitative Biology Center (QBiC), University of Tübingen, Tübingen, Germany.; ^17^ Department of Biomedical Sciences, Seoul National University Graduate School, Seoul, Republic of Korea.; ^18^ Cancer Research Institute, Seoul National University College of Medicine, Seoul, Republic of Korea.; ^19^ PB Immune Therapeutics Inc., Seoul, Republic of Korea.; ^20^ Liver Center, University of California San Francisco, San Francisco, CA 94143, USA.; ^21^ Department of Pathology, University of California San Francisco, San Francisco, CA 94143, USA.; ^22^ Eli and Edythe Broad Center of Regeneration Medicine and Stem Cell Research Department of Pathology Liver Center, University of California San Francisco, San Francisco, CA 94143, USA.; ^23^ Department of Microbiology and Immunology, University of California San Francisco, San Francisco, CA 94143, USA.; ^24^ Howard Hughes Medical Institute, University of California San Francisco, San Francisco, CA 94143, USA.; ^25^ Department of Medicine, Infectious Diseases Division, University of California San Francisco, San Francisco, CA 94143, USA.; ^26^ Diabetes Center, University of California San Francisco, San Francisco, CA 94143, USA.

## Abstract

Fibroblasts are dynamic structural cells that direct both beneficial tissue repair and pathological organ fibrosis through interactions with tissue-resident type 2 lymphocytes (T2Ls) and type 3/17 lymphocytes (T3Ls). The cytokines interleukin-13 (IL-13) and IL-17A, produced by T2Ls and T3Ls, respectively, are linked to both tissue inflammation and fibrosis, but how their spatial positioning influences beneficial or pathological organ remodeling remains unclear. Using mouse models of liver injury and fibrosis, three-dimensional microscopy, and spatial transcriptomics, we found an accumulation of periportal and fibrotic tract T2Ls, predominantly group 2 innate lymphoid cells (ILC2s), positioned near T3Ls and niche adventitial fibroblasts and adjacent to discrete profibrotic myofibroblasts. Unexpectedly, T2L ablation worsened both carbon tetrachloride– and bile duct ligation–induced liver fibrosis, accompanied by increased IL-17A^+^ T3Ls, predominantly γδ T cells. In contrast, concurrent T2L and T3L ablation reduced liver fibrosis. Our work suggests a spatially associated cross-talk between liver lymphocytes and fibroblast niches that tunes liver repair but can go awry in pathological liver fibrosis.

## Teaser

T2Ls and T3Ls colocalize in liver fibroblast niches; T2L loss drives T3L expansion and worsens fibrosis.

## INTRODUCTION

Fibrotic diseases are widespread across organs, increase with age, and are major drivers of global morbidity and mortality, with particularly severe consequences in the liver (*1*). Chronic liver (hepatic) injury and inflammation, driven by viral infections (e.g., hepatitis B or C); obstruction or impairment of bile drainage (e.g., gallstones and liver/biliary/pancreatic cancer); or obesity-associated “metabolic dysfunction–associated steatohepatitis” (MASH; formally called NASH) each increases the risk of liver cirrhosis and downstream hepatocellular carcinoma (*2*). Fibrogenic and inflammatory pathways synergize to regulate liver fibrosis through activation of resident liver stromal cells [e.g., hepatic stellate cells and periportal adventitial fibroblasts (AFs)], promoting the development of pathogenic, matrix-depositing myofibroblasts (MFs); long-term liver scarring; and loss of hepatic function.

Tissue-resident lymphocytes are critical regulators of tissue inflammation, secreting cytokines that act on other immune cells such as macrophages, as well as directly on tissue cells such as epithelial, endothelial, and stromal cells to coordinate discrete aspects of antimicrobial immunity and tissue remodeling (*3*, *4*). They are often categorized into three groups with associated canonical cytokines, herein called type 1 lymphocytes (T1Ls), T2Ls, and T3Ls. These groups share upstream regulatory signals, transcriptional programs, and effector function across CD4^+^ helper T cells [T helper 1 (T_H_1), T_H_2, and T_H_17 cells], innate lymphoid cells [group 1 innate lymphoid cells (ILC1s), ILC2s, and ILC3s], and subsets of innate-like T cells [γδ, nuclear killer T (NKT), and mucosal-associated invariant T (MAIT) cells]. Although plasticity exists, and lymphocytes do not always fit precisely, these categories remain functionally useful and clinically relevant constructs to define temporal and spatial patterns of tissue immunity (*5*).

Among these, T3Ls that secrete IL-17A^+^ and IL-22^+^ and T2Ls (ILC2s and T_H_2 subsets) that secrete interleukin-4 (IL-4), IL-5, IL-9, and IL-13 (*6*–*14*) have both been implicated in fibrotic and inflammatory processes. T3L responses, while important for tissue regeneration, can drive fibrosis when excessive, often mediated by IL-17A and/or IL-22 acting on diverse nonhematopoietic cell types (*15*–*20*). T2L responses, regulated by upstream signals such as IL-33, can similarly promote fibrosis via IL-4 and IL-13 (*21*–*26*). However, type 2 immunity can also promote beneficial tissue development, remodeling, and repair (*27*, *28*). ILC2s in particular are implicated in both chronic inflammatory diseases and helminth-driven fibrosis (*24*, *29*, *30*), but their role in liver fibrosis outside type 2–skewed contexts remains unclear.

In multiple tissues, T2Ls are enriched in collagen-dense fibroblast–laden regions of larger vessels and other border or fascial structures, including periportal liver regions (*31*, *32*). Perivascular adventitial regions are defined by a fibroblast state that produces IL-33 and thymic stromal lymphopoietin (TSLP), designated AFs, that directly support T2Ls (*33*, *34*). However, additional immune subsets, including regulatory T cells (T_reg_ cells), dendritic cells, and interstitial macrophages, are enriched in the adventitia, suggesting that these may be sites that coordinate regional tissue immunity, particularly after inflammation ensues (*32*). AFs are present in all organs as a universal fibroblast state with mesenchymal progenitor capacity and the ability to differentiate into a pathogenic MF state that can drive organ fibrosis (*35*). T3Ls are enriched within a range of lymphoid tissues in the intestine and tertiary lymphoid tissues/structures, although their global tissue topography is not well defined. Together, the cooperative functions of immune-interactive adventitial-like fibroblasts, profibrotic MFs, and liver lymphocytes during liver fibrosis are unclear.

Here, we demonstrate that liver fibrosis, driven in the toxin carbon tetrachloride (CCl_4_) or bile duct ligation (BDL) models, promotes the expansion of IL-33^high^ immune-interactive adventitial-like fibroblasts in proximity to expanded T2Ls (predominantly ILC2s) and T3Ls (IL-17A^+^ RORγt^+^, predominantly γδ T cells) in liver periportal adventitial regions, as well as newly deposited fibrotic tracts. We show that AF-like fibroblasts are concentrated in periportal and fibrotic regions, as well as near cellularly distinct but less abundant MFs. Using selective ablation of IL-5^+^ lymphocytes, loss of T2Ls exacerbated liver fibrosis in both models, associated with increased T3Ls. Co-depletion of T2Ls and T3Ls attenuated CCl_4_-induced hepatic fibrosis and liver inflammation. We hypothesize that key conversations between T2Ls and T3Ls occur in specialized immune-interactive fibroblast niches that expand during liver fibrosis and ultimately tune the degree of chronic liver damage and fibrosis.

## RESULTS

### Mapping stromal cell topography in liver fibrosis

**Fig. 1.**
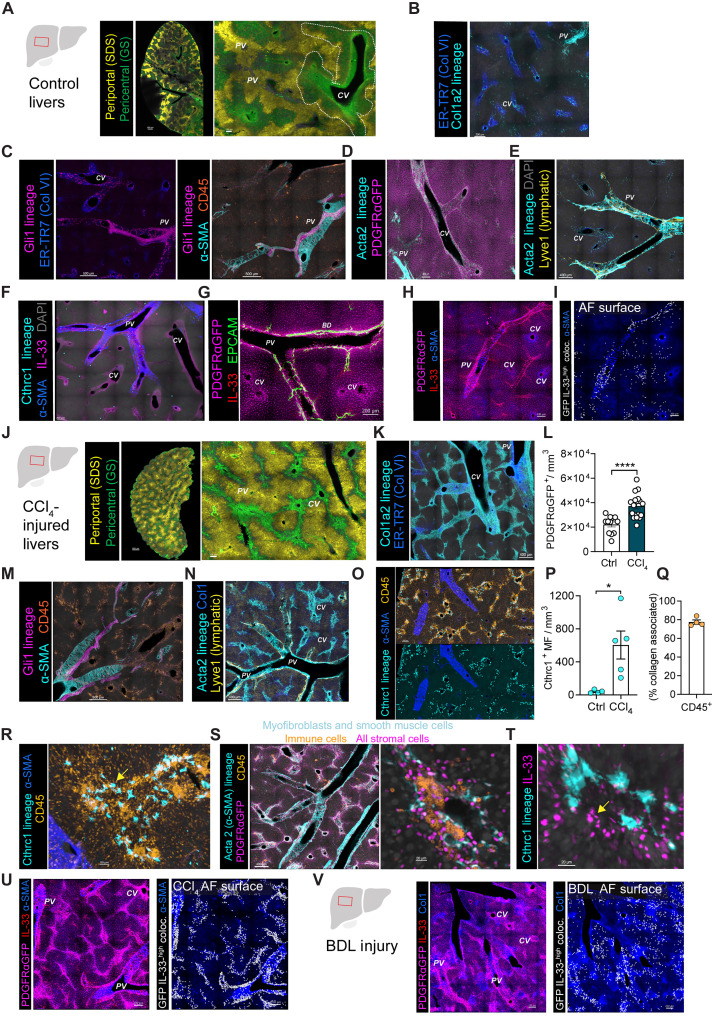
Mapping stromal cell 3D topography in liver fibrosis. (**A**) Confocal liver image from IL-33^mcherry/+^ mice stained for GS and sodium dodecyl sulfate (SDS). PV (zone 1) and CV (zone 3). *n* ≥ 3 mice. Scale bars, 1000 μm. (**B**) Confocal image from Col1a2CreERT; R26-TdT^+^ livers. *n* ≥ 3. Scale bar, 200 μm. (**C**) Confocal images from Gli1 lineage tracker mice. Scale bars, 500 μm. (**D**) Confocal images from α-SMA-creERT2; PDGFRαGFP mice. *n* ≥ 2. Scale bar, 300 μm. (**E**) Confocal images from aSMA-creERT2 mice. *n* ≥ 2. Scale bar, 400 μm. (**F** and **G**) Confocal images from Cthcr1-creER mice (F) and PDGFRαGFP; IL-33^mcherry/+^ mice (G). *n* ≥ 3. Scale bar, 200 μm. (**H** and **I**) Confocal images from naïve PDGFRαGFP; IL-33^mcherry/+^ livers (H) and PDGFRαGFP-IL-33^mcherry^ colocalization (I). *n* ≥ 3. Scale bar, 200 μm. (**J**) Confocal image from 4-week-old CCl_4_-treated IL-33^mcherry/+^ mice. *n* ≥ 3. Scale bars, 1000 μm. (**K**, **M**, and **N**) Confocal images from 4-week-old CCl_4_-treated Co1a2CreERT; R26-TdT^+^, Gli1 lineage tracker, and α-SMA-creERT2 mice. *n* ≥ 3 per group. Scale bars, 400 μm. (**L**) Quantification of PDGFRαGFP^+^ cells per tissue volume in PDGFRαGFP; Il5tdtomato-^Cre/+;^ Rosa26^RFP/+^ mice ± CCl_4_. *n* = 3 to 5 mice per group. (**O**, **P**, **R**, and **T**) Confocal images and quantification of Cthrc1^+^ MFs per tissue volume in 4-week-old CCl_4_-treated Cthrc1CreERT2; R26-TdT lineage tracker mice. *n* ≥ 3 per group. Scale bars, 50 μm (R) and 20 μm (T). (**Q**) CD45^+^ leukocytes <25 μm from Col1a2creERT2; R26-TdT lineage-traced cells. *n* = 4, two experiments. (**S**) Confocal images from 4-week-old CCl_4_-treated Acta2CreERT2; R26-TdT; PDGFRαGFP mice. *n* ≥ 2. Scale bars, 200 μm (left) and 20 μm (right). (**U** and **V**) Confocal images from PDGFRαGFP; IL-33^mcherry/+^ mice after 4-week CCl_4_ treatment (U) or 2-week BDL (V), with colocalization highlighted. *n* ≥ 3 per group. Scale bars, 200 μm. Bar graphs indicate the means (±SE). *P ≤ 0.05 and ****P ≤ 0.0001. Student’s *t* test (L and P). See also fig. S1.

The liver has a unique organ macrostructure, with portal triads composed of portal veins (PVs) delivering nutrient-rich venous blood from the small intestine, hepatic arteries carrying oxygenated blood, and biliary ducts draining from the liver to the small intestine. Blood percolates from portal triads through the liver parenchyma, defined by hepatocytes, porous sinusoidal endothelium, and hepatic stellate cells (HSCs; e.g., liver mural cells/pericytes), and is ultimately collected in hepatic central veins (CVs; Fig. 1A). Using thick-section three-dimensional (3D) confocal microscopy, we first determined the topography of all liver fibroblasts [collagen 1 (Col1) lineage-traced; Co1a2CreERT; R26-TdT^+^] in resting liver, finding that fibroblasts were sparse and predominantly localized to periportal adventitial regions and less abundantly at pericentral regions (Fig. 1B). Immune cells were broadly distributed throughout the liver, with occasional small clusters identified in proximity to liver AFs in PV or CV regions (CD45^+^; Fig. 1C).

Fibroblasts have discrete in vivo states driven by fibroblast positioning, immune cell interactions, and fibroblast function. AFs represent one such conserved fibroblast state that localizes to tissue large-vessel boundaries, fascial planes, and organ surfaces and defines a shared cross-tissue border niche for T2Ls (*31*, *32*). Subsets of AFs have high mesenchymal progenitor/stem-cell capacity and are marked by the expression of the hedgehog effector Gli1 (*32*, *36*, *37*). Using a Gli1-lineage tracker mouse strain (Gli1CreERT2; R26-fsf-TdT), Gli1^TdT+^ stromal cell topography was similar to that of all Col1^TdT+^ fibroblasts (Fig. 1B), with enrichment at adventitial PV and PC regions (Fig. 1C) and often near smooth muscle cells (Acta2CreERT2; R26-fsf-TdT; Fig. 1, D and E). After tissue injury, a profibrotic fibroblast state emerges, here called MFs. To more precisely chart MF liver topography, we used mouse strains that labeled cells in the MF state as well as any arising cellular progeny (Cthrc1CreERT2; R26-TdT and Acta2CreERT2; R26-fsf-TdT^+^) (*38*, *39*). As expected, bona fide MF state fibroblasts were quite sparse in resting livers, with scant labeling of a periportal stromal subset (Fig. 1, D to F). Last, we used a pan-stromal cell reporter (PDGFRαGFP) to confirm the broad distribution of resting liver stromal cells, marking both AFs and parenchymal-localizing HSCs (Fig. 1G).

Many liver stromal cells coexpressed high levels of the cytokine and alarmin IL-33 (IL-33^mcherry^) (Fig. 1, H and I), a conserved feature of AFs and other border fibroblasts that we have previously characterized across organs (*31*, *32*, *36*, *40*); however, some parenchymal-associated stromal cells (PDGFRαGFP^variable^) also expressed low levels of IL-33^mcherry^ (movie S1). Flow cytometry confirmed that these cells were HSCs (Retinol^+^ FSC^low^ GFP^variable^) that expressed low IL-33 compared to liver AFs (fig. S1, A and B), consistent with previous single-cell RNA sequencing results (fig. S1C) (*41*). Liver venous endothelial cells and rare epithelial subset(s) also expressed variable IL-33^mcherry^ (fig. S1D).

Next, we used two well-established models of liver injury and subsequent fibrosis. (i) CCl_4_ repetitive dosing caused hepatocyte injury and centrilobular liver fibrosis (fig. S1E) (*42*), liver damage [elevated serum alanine aminotransferase (ALT)], collagen deposition and fibrosis (hydroxyproline levels and Sirius red staining), leukocytosis, and loss of pericentral hepatocytes [decreased glutamine synthetase (GS)] (fig. S1, E to I and O) (*43*). (ii) BDL is a surgical model that caused acute obstructive jaundice with cholestasis-induced liver fibrosis focused in periportal bile duct–containing regions (fig. S1J) (*44*). At 14 days post-BDL, mice had increased liver damage, cholestasis, liver collagen deposition at periportal bile duct–containing regions, and liver fibrosis (fig. S1, K to O).

To map liver fibroblast topographic positioning after injury, we first focused on the CCl_4_ repetitive injury model with pericentral liver damage and downstream fibrosis (Fig. 1J). Liver total stromal cells increased in pericentral regions and bridging fibrosis tracts that spanned pericentral to periportal zones (Fig. 1, K and L). As expected, Gli1^TdT+^ AFs remained confined to adventitial periportal and pericentral regions (Fig. 1M), consistent with an expected ontogeny from HSCs to MFs in the CCl_4_ model (*40*); in contrast, periportal AFs expanded and more markedly contributed in periportal and cholestatic fibrosis, including in BDL mouse models (*45*). As expected, CCl_4_-driven MFs (Acta2^TdT+^ lineage^+^ fibroblasts) expanded throughout fibrotic regions, predominantly in pericentral and bridging fibrosis zones (Fig. 1N). Using the more specific MF state mouse tracker (Cthrc1-lineage^+^), we found the predicted increase in liver MF density with a topography similar to Acta2^TdT+^ MFs (Fig. 1, O and P). Flow cytometry confirmed that MFs (Acta2^RFP+^, PDGFRαGFP^variable^) were elevated in fibrotic livers (fig. S1P). Whereas MFs are traditionally held to be profibrotic and immune-exclusionary/suppressive (*46*), increased immune cells predominantly colocalized within fibroblast-dense regions (~75%; marked by Col1a2^TdT+^) (Fig. 1, Q to S), raising the question of fibroblast heterogeneity within these regions of fibrosis.

In contrast to the profibrotic MF state, we and others have described a cross-organ AF border state that can be immune-supportive, acting via multiple secreted and contact-dependent mechanisms (*31*, *32*, *36*, *39*). As such, we asked whether AF-like liver fibroblasts may also be present near immune cells in emerging areas of fibrosis. Using flow cytometry, we confirmed that AF-like fibroblasts were elevated (IL-33^mcherry+^; fig. S1Q) and were largely distinct from α-SMA^+^ MFs (fig. S1R). Microscopy confirmed that putative AFs (PDGFRαGFP^+^ IL-33^high^) were in proximity to MFs but were more numerous in both periportal regions and along de novo fibrotic tracts associated with CVs and bridging fibrosis (Fig. 1, T and U; fig. S1S; and movie S2). BDL surgery also expanded periportal AF-like fibroblasts (Fig. 1V). Together, these data indicate that AF-like fibroblasts are enriched in periportal and de novo perifibrotic regions and in proximity to cellularly discrete, yet more sparse, MFs (*47*), suggesting that emerging fibrotic tracks represent key sites where two discrete fibroblast states coexist and may link liver inflammation and fibrosis.

### T2Ls and T3Ls expand with liver damage and fibrosis

**Fig. 2.**
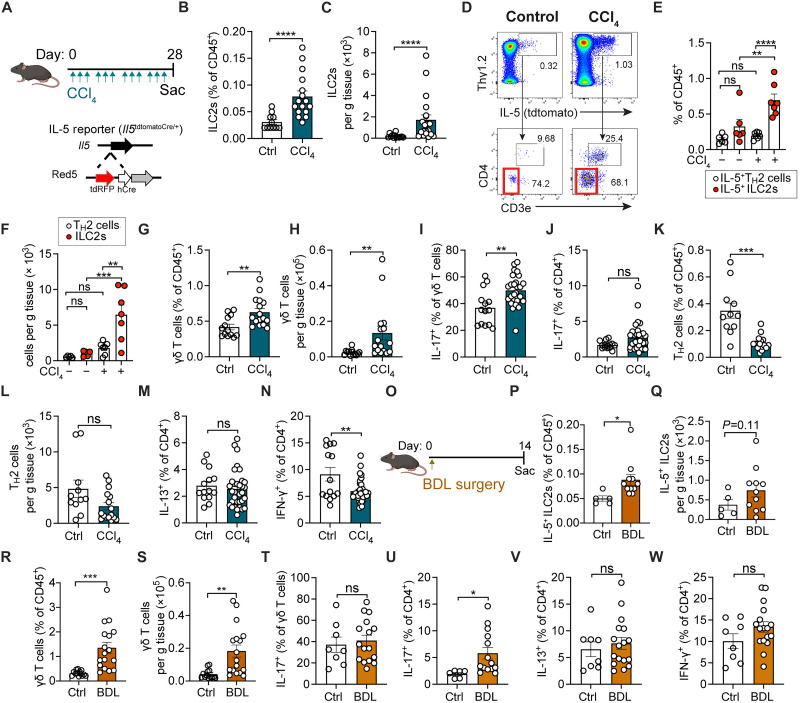
ILC2s and γδ T cells expand in different models of liver fibrosis. (**A**) Schematic showing CCl_4_ administration in IL-33^mcherry/+^ reporter mice, 0.5 μl CCl_4_/g body weight (BW) ip, three times per week for 4 weeks (4w), relevant to (B) to (N). Pooled data from three independent experiments; *n* ≥ 4 mice per group, unless otherwise noted. (**B** and **C**) Flow cytometry showing percentages (B) and total numbers (C) of ILC2s in livers from vehicle (corn oil)– or CCl_4_-treated mice. (**D** to **F**) Flow cytometry plots (D) and quantification [(E) and (F)] of hepatic IL-5^+^ lymphocytes gated on CD45^+^CD11b^−^CD19^−^NK1.1^−^Thy1.2^+^ from Il5-tdtomato-Cre mice, highlighting ILC2s (IL-5^+^CD3^−^CD4^−^) and T_H_2 cells (IL-5^+^CD3^+^CD4^+^). Total *n* ≥ 3 mice per group. ns, not significant. (**G** to **N**) Percent (G) and total γδ T cells (H), percent IL-17^+^ γδ T cells (I) and IL-17^+^ CD4^+^ T cells (J), percent (K) and total T_H_2 cells (L), percent IL-13^+^CD4^+^ T cells (M), and IFN-γ^+^CD4^+^ T cells (N) in livers from vehicle- or CCl_4_-treated IL-33^mcherry/+^ mice. (**O**) Schematic of BDL in IL-5^+^ lymphocyte lineage tracker mice (IL-5tdtomato-Cre; Rosa26RFP). (**P** to **S**) Percent (P) and total liver IL-5^+^ ILC2s (Q), percent (R) and total liver γδ T cells (S) 14 days post-BDL or post–sham surgery, pooled from two experiments; *n* ≥ 2 mice per group. (**T** to **W**) Percent IL-17^+^ γδ T cells (T), IL-17^+^ CD4^+^ T cells (U), IL-13^+^CD4^+^ T cells (V), and IFN-γ^+^CD4^+^ T cells (W) in livers from C57BL/6 mice 14 days post-BDL or postsham, pooled from three independent experiments; *n* ≥ 2 mice per group. Bar graphs indicate the means (±SE). Student’s *t* test [(B), (C), (G) to (N), and (P) to (W)] or one-way ANOVA with Tukey post test [(E) and (F)]; *P ≤ 0.05, **P ≤ 0.01, ***P ≤ 0.001, and ****P ≤ 0.0001. See also fig. S2.

We have previously shown that AF state fibroblasts support T2Ls but are also spatially associated with a range of resident immune cells. To define potential relationships between AF-like liver fibroblasts and immune cells, we first quantified liver immune cells via flow cytometry. Liver ILC2s increased with CCl_4_ fibrosis (Fig. 2, A to C; gating in fig. S2A), similar to human patients with MASH and associated fibrosis (*30*); in contrast, blood ILC2 levels were unchanged (fig. S2B). To further characterize ILC2s, we used well-defined mouse IL-5 cytokine reporters, often in combination with lineage tracker/fate mapper alleles (IL-5-TdT-Cre; R26-fsf-TdT) (fig. S2C) (*31*, *32*). ILC2s were 70 to 80% of the IL-5^TdT+^ lineage-tracked liver cells, with the remainder identified as T cells that included both CD4^+^ T_H_2 and innate-like T cells (CD3e^+^CD4^−^CD8^−^) (Fig. 2, D to F). Both ILC2s and IL-5^+^ T_H_2 cells increased with CCl_4_ liver fibrosis; however, ILC2 (lin^−^ thy1^+^ Arg1^+^) expression of IL-5 (TdT), IL-13 (hCD4), and CD25 (IL-2Ra) was not altered (fig. S2, D to H), suggesting T2L expansion without overt cytokine activation.

To determine whether the liver fibrosis–associated expansion of liver T2Ls (IL-5^TdT+^ lymphocytes) was unique, we profiled liver immune cells more broadly. During CCl_4_ fibrosis, liver CD4^+^ and CD8^+^ T cells, natural killer (NK) cells, and neutrophils expanded with relatively unchanged proportions (fig. S2, I to M). However, γδ T cells and their IL-17A expression, as well as T_H_17 cells, also increased (Fig. 2, G to J). In contrast, relative proportions and total T_H_2 cells (defined as CD3e^+^ CD4^+^ FoxP3^−^ Gata3^hi^; Fig. 2, K to M) or T_H_1 cells (Fig. 2N) remained unchanged or decreased. T_reg_ cells were also elevated (fig. S2, K and L), commonly observed in chronic inflammatory settings (*48*). The BDL model of liver fibrosis was also associated with increased IL-5^+^ ILC2s (Fig. 2, O to Q), γδ T cells (Fig. 2, R and S), and neutrophils at 14 days postsurgery (fig. S2N), with a relative increase in adaptive T_H_7 cells, whereas T_H_2 cells, T_H_1 cells, and the proportion of γδ T cells that expressed IL-17A remained unchanged (Fig. 2, T to W). CD4^+^ and CD8^+^ T cells were increased but proportionally unchanged after BDL surgery (fig. S2, O and P). Together, these results suggest a conserved liver fibrosis–associated expansion of T2Ls and T3Ls, at least in the two well-established mouse models of liver fibrosis used.

### Liver IL-5^+^ T2Ls localize to periportal regions of control livers and expand in fibrotic tracts

**Fig. 3.**
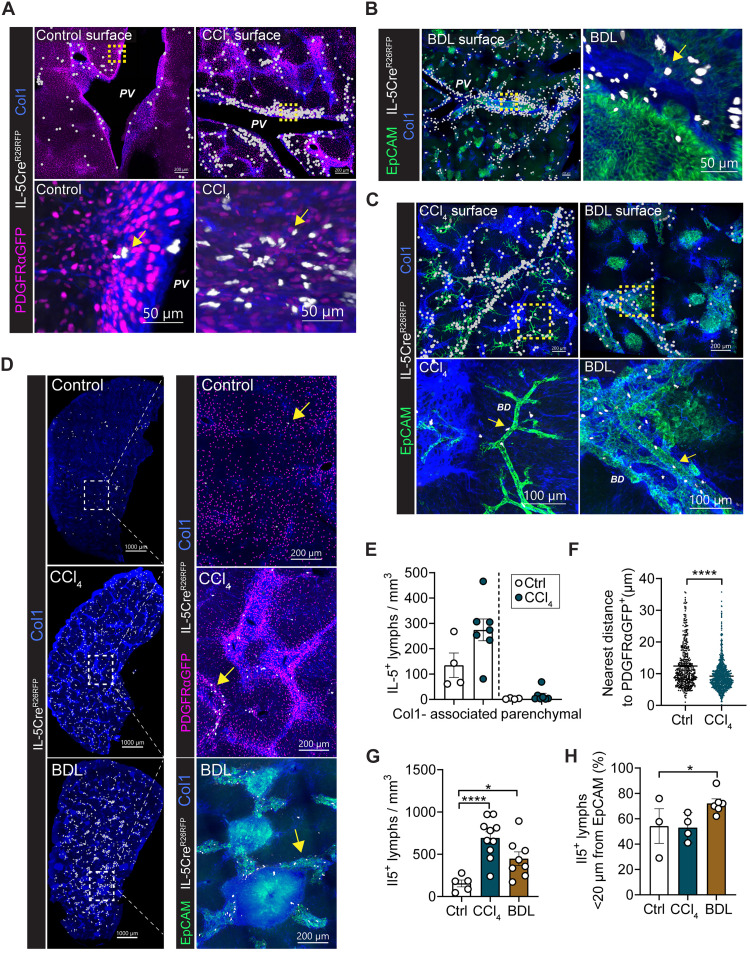
Liver IL-5^+^ T2Ls localize to periportal regions of control livers and expand in fibrotic tracts. (**A**) Representative thick section confocal or 3D-rendered images from PDGFRαGFP; Il5tdtomato-^Cre/+;^ Rosa26^RFP/+^ mice at rest or after 4 weeks of CCl_4_ treatment (0.5 μl CCl_4_/g BW ip, three times per week; see fig. S1E). Sections (200 μm coronal) were stained for Col1. Images represent *n* = 3 or 4 mice per group (both sexes, different littermates), with two or three thick sections per mouse. Yellow arrows mark IL-5^+^ lymphocytes; PV (zone 1). Scale bars, 200 μm, unless otherwise indicated. (**B**) Representative confocal or 3D-rendered images from PDGFRαGFP; Il5tdtomato-^Cre/+;^ Rosa26^RFP/+^ mice at rest or 14 days post-BDL (see fig. S1J). Sample preparation as in (A); *n* = 3 or 4 mice per group. Yellow arrows indicate IL-5^+^ lymphocytes. Scale bars, 200 μm unless otherwise indicated. (**C**) Representative confocal liver tissue sections and surface analysis from IL-5^+^ lymphocyte lineage tracker mice (IL-5tdtomato-Cre; Rosa26RFP) after 4-week CCl_4_ treatment or 14 days post-BDL. *n* ≥ 3 mice per group. Yellow arrows indicate IL-5^+^ lymphocytes; BD, bile duct. Scale bars, 200 μm unless otherwise indicated. (**D**) Confocal images (200 μm thick) showing IL-5^+^ lymphocytes (RFP^+^) and PDGFRαGFP^+^ fibroblasts in control, 4-week CCl_4_–treated, or BDL livers. *n* ≥ 3 mice per group. Yellow arrows mark IL-5^+^ cells. Scale bars, 1000 μm (left) and 200 μm (right). (**E**) Quantification of Col1-associated (<60 μm) and parenchymal (>60 μm) IL-5^+^ lymphocytes per tissue volume. *n* = 4 to 7 mice per group. (**F**) Quantification of IL-5^+^ lymphocytes to the nearest PDGFRαGFP^+^ fibroblast. *n* = 3 to 5 mice per group. (**G**) IL-5^+^ lymphocytes per tissue volume. *n* = 2 or 3 mice per group. (**H**) Percent IL-5^+^ lymphocytes <20 μm from EpCAM^+^ epithelial duct cells. *n* = 2 to 6 mice per group. Bar graphs indicate the means (±SE), Student’s *t* test (F) or one-way ANOVA with Tukey post test [(G) and (H)]. *P ≤ 0.05 and ****P ≤ 0.0001. See also fig. S3.

Next, we used volumetric confocal microscopy to determine the positioning of liver T2Ls, including their spatial relationship to fibroblast subsets/states. In naïve livers, IL-5^+^ T2Ls were relatively rare and enriched near fibroblasts in periportal and collagen-dense adventitial networks, often near bile ducts and hepatic arteries, as we have previously described (*31*, *32*); after CCl_4_ injury, IL-5^+^ T2Ls increased in density, localizing in collagen-dense adventitial regions (i.e., periportal veins and CVs) as well as along de novo fibrotic tracts (Fig. 3A; fig. S3, A and B; and movies S3 to S5). Even at rest, IL-5^+^ T2Ls were readily identified in peribiliary regions (fig. S3C); however, T2Ls also expanded after BDL surgery and continued to localize in expanded collagen-dense regions near portal bile ducts, concomitant with ductal proliferation and portal fibrosis (Fig. 3, B to D, and fig. S3D). Although already in close proximity, T2Ls expanded and localized more tightly with fibroblasts (PDGFRαGFP^+^) after CCl_4_-induced fibrosis (Fig. 3, E and F). BDL and CCl_4_ both drove increased T2Ls, although BDL preferentially increased T2L accumulation near bile ducts (EpCAM^+^; Fig. 3, G and H). To better understand whether this T2L expansion was a conserved feature of fibrosis in other organs, we compared our results to those of a bleomycin model of lung injury/fibrosis (fig. S3, E and F) (*49*). Lung IL-5^+^ lymphocytes also expanded within collagen-dense adventitial and de novo fibrotic lung regions but did not expand in the blood (fig. S3, G to I). Together, these data indicate that T2Ls primarily reside in collagen-dense liver adventitial regions as well as de novo fibrotic tracts in close proximity to subset(s) of expanded adventitial-like fibroblasts.

### T3L liver topography

**Fig. 4.**
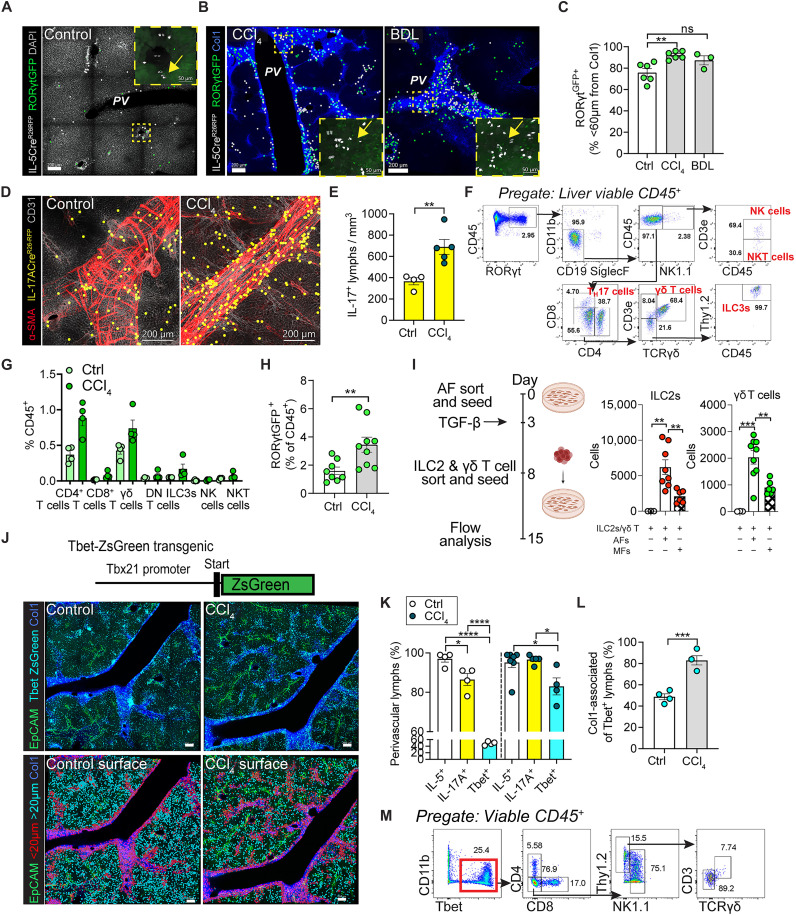
T3Ls have similar liver topography to IL-5^+^ T2Ls. (**A** and **B**) Thick section confocal imaging from livers of RORγtGFP; Il5tdtomato-^Cre/+;^ Rosa26^RFP/+^ mice at the steady state (A) or after 4-week CCl_4_ treatment or 14 days post-BDL (B). Higher magnification shows RORγtGFP^+^ cells near IL-5^+^ lymphocytes (yellow arrows). PV (zone 1). Images represent two experiments; *n* = 7 or 8 mice per group. (**C**) Quantification of RORγt^+^ cells <60 μm from Col1; *n* ≥ 3 mice per group. (**D**) Confocal imaging of control and 4-week CCl_4_–treated IL-17A lineage tracker mice (Il17atdtomato-^Cre/+;^ Rosa26^RFP/+^). *n* ≥ 4 mice per group. (**E**) IL-17^+^ lymphocytes per tissue volume. *n* = 4 or 5 mice per group. (**F**) Flow gating scheme for liver RORγtGFP^+^ cells from reporter mice. (**G**) Quantification of RORγtGFP^+^ subsets among CD45^+^ leukocytes in vehicle- or CCl_4_-treated mice. *n* ≥ 4 mice per group. (**H**) Percent RORytGFP^+^ liver cells from reporter mice ± 4-week CCl_4_ treatment; pooled from two experiments; *n* ≥ 9 mice per group. (**I**) PDGFRα^+^Sca1^+^ lung AFs cultured for 6 to 7 days with lung ILC2s and γδ T cells; TGF-β added to induce MF differentiation. *n* = 3 experiments. (**J**) Tbx21-ZsGreen “Tbet” reporter schematic, relevant to (J) to (M), and representative liver image after 4-week CCl_4_ treatment or vehicle. Two mice per group, two sections each. (**K**) Perivascular IL-5^+^, IL-17A^+^, and Tbet^+^ lymphocytes: total; periportal <60 μm from α-SMA^+^, Col1a^low^ or Col1a^−^, GS^−^; and pericentral <60 μm from α-SMA^low^, Col1a^+^, GS^+^. *n* = 4 to 6 mice per group. (**L**) Percent Tbet^+^ cells <60 μm from Col1. *n* = 2 to 4 mice per group. (**M**) Flow gating scheme for liver ZsGreen expression in Tbet-ZsGreen mice after 4-week CCl_4_ treatment. All scale bars, 200 μm. Bar graphs indicate the means (±SE), Student’s *t* test [(E), (H), and (L)] or one-way ANOVA with Tukey post test [(C) and (K)]. *P ≤ 0.05, **P ≤ 0.01, ***P ≤ 0.001, and ****P ≤ 0.0001.

As IL-17A–producing T3Ls, including γδ T cell subsets and CD4^+^ T_H_17 cells, were also elevated in our models of hepatic fibrosis, we determined their liver topography using RAR-related orphan receptor γt (RORγt)-green fluorescent protein (GFP) (*50*) or IL-17A lineage tracker mice (IL-17Cre; R26-fsf-TdT) (*51*). In resting livers, T3Ls resided predominantly in adventitial periportal areas, similar to T2Ls (Fig. 4, A, C, and K). After induction of fibrosis with CCl_4_ or BDL, T3Ls further accumulated in collagen-dense areas of the adventitia, as well as along de novo fibrotic tracts, observed in proximity to IL-5^+^ T2Ls (Fig. 4, B to E and K). Flow cytometric analysis demonstrated that liver RORγt-GFP^+^ T3Ls were composed of γδ T cells and CD4^+^ T_H_17 cells, with further minor subsets including other innate-like T cells and ILC3s (Fig. 4, F and G); RORγt-GFP^+^ T3Ls increased with CCl_4_ fibrosis, predominantly composed of γδ T cells and T_H_17 cells (Fig. 4, G and H). To test whether AFs were sufficient to support both T2Ls and T3Ls, we sorted primary lung AFs and cocultured them with sort-purified lung ILC2s or γδ T cells. AF cocultures were sufficient to maintain both ILC2s and γδ T cells in the absence of cytokine supplement or T cell receptor (TCR) stimulation (Fig. 4I), as shown for ILC2s and IL-5^+^ T_H_2 cells (*32*). In contrast, AFs preconditioned with transforming growth factor–β (TGF-β) to drive an MF state had impaired ILC2 and γδ T cell support (Fig. 4I). We conclude that AFs are sufficient to support both T2Ls (ILC2s/T_H_2 cells) and T3Ls (γδ T cells); furthermore, liver AF–like fibroblast expansion during fibrotic challenge was spatially and temporally associated with increased T2Ls and T3Ls.

To compare with T1Ls, we next performed thick-section microscopy on T1L reporter mice [Tbx21-ZsGreen, transcription factor associated with lymphocytes that can produce interferon-γ (IFN-γ)] (*32*). In contrast to T2Ls and T3Ls, T1Ls and IFN-γ are linked to protection from MASH and liver fibrosis (*52*). At rest, T1Ls (Tbet-ZsGreen^+^) were more abundant than T2Ls or T3Ls, similar to other tissues, and were broadly distributed across liver sinusoidal and collagen-dense adventitial regions (*31*) with some enrichment near pericentral hepatic veins (Fig. 4J). CCl_4_-induced liver injury drove modest T1L expansion and localization to collagen-rich regions, albeit less than T2Ls or T3Ls (Fig. 4, J to L). Flow cytometry confirmed that naïve liver T1Ls were a mixture of innate T1Ls (NK cells and ILC1s) and subsets of adaptive CD4^+^ and CD8^+^ T cells and innate-like T cells (Fig. 4M). These data suggest that the distribution of liver T1Ls is distinct from T2Ls and T3Ls, both at rest and in liver fibrosis driven by CCl_4_, with a relative type 1 preference for liver parenchymal and CV-associated regions (*31*).

### Loss of IL-5^+^ T2Ls exacerbates hepatic fibrosis

**Fig. 5.**
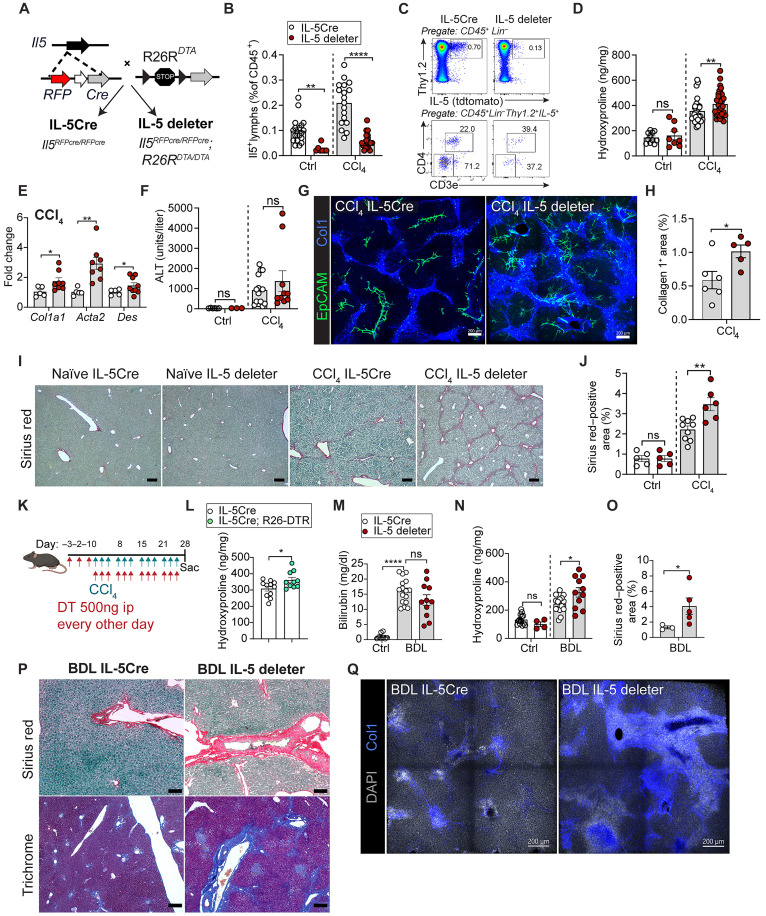
Loss of IL-5^+^ T2Ls exacerbates hepatic fibrosis. (**A**) Schematic of IL-5–deleter mice (*Il5*^Cre-RFP/Cre-RFP^*; R26R*^DTA/DTA^). IL-5 deleters were homozygous for *R26R^DTA^*, and controls lacked DTA. (**B**) Flow quantification of liver IL-5^+^ lymphocytes from control or CCl_4_-treated Il5-tdtomato-Cre mice and IL-5–deleter mice. Pooled from three independent experiments; *n* ≥ 4 mice per group. (**C**) Flow plots showing IL-5^+^ lymphocytes (Lin^−^Thy1.2^+^Red5^+^), IL-5^+^ ILC2s (Lin^−^Thy1.2^+^CD3e^−^CD4^−^), and IL-5^+^ T_H_2 cells (Lin^−^Thy1.2^+^CD3e^+^CD4^+^) in livers from IL-5tdtomato-Cre or IL-5–deleter mice after 4-week CCl_4_ treatment. (**D**) Liver hydroxyproline in controls or 4-week CCl_4_–treated Il5-tdtomato-Cre and IL-5–deleter mice. Pooled from three experiments. *n* ≥ 8 per group. (**E**) Liver expression of *Col1a1*, *Acta2*, *and Des*, normalized to *GAPDH* in control or 4-week CCl_4_–treated Il5-tdtomato-Cre and IL-5–deleter mice (*n* ≥ 5 mice per group, two experiments). (**F**) ALT levels in same groups (*n* ≥ 8 mice per group). (**G**) Confocal liver sections in control or 4-week CCl_4_–treated Il5-tdtomato-Cre mice and IL-5–deleter mice (*n* = 3 or 4 per group). (**H**) Quantification of Col1^+^ area per tissue volume after 4-week CCl_4_ (*n* = 2 to 4 mice per group). (**I** and **J**) Sirius red staining (I) and quantification (J) in IL-5–deleter and control mice ± CCl_4_ (*n* ≥ 5 mice per group). (**K**) Schematic of CCl_4_ and DT regimen in IL-5^DTR^ (*Il5*^Cre-RFP/Cre-RFP^*; R26R*^DTR/DTR^) and control mice. (**L**) Hydroxyproline in IL-5tdtomato-Cre and IL-5^DTR^ mice after 4-week CCl_4_ treatment (*n* ≥ 5 mice per group). (**M** and **N**) Bilirubin (M) and hydroxyproline (N) in IL-5–deleter and control mice 14 days post-BDL (*n* ≥ 4 mice per group, three experiments). (**O** and **P**) Sirius red [(O) and (P)] and trichrome (P) staining 14 days post-BDL (*n* ≥ 4 mice per group). (**Q**) Confocal liver sections 14 days post-BDL. All scale bars, 200 μm. Bar graphs indicate the means (±SE). Student’s *t* test [(B), (D) to (F), (H), (J), and (L) to (O)]. *P ≤ 0.05, **P ≤ 0.01, and ****P ≤ 0.0001. See also fig. S4.

Next, we tested the function of T2Ls during the development of liver fibrosis. Given the involvement of IL-13 in type 2 immune-driven tissue fibrosis (*21*), we hypothesized that T2L deficiency would offer protection from liver fibrosis. Using IL-5–deleter mice (“T2L deleters”), a well-established genetic approach that sensitively and specifically eliminates IL-5–expressing cells via induced diphtheria toxin (DT) expression, including most ILC2s and rare IL-5^+^ T_H_2 cells (*Il5*^Cre-RFP/Cre-RFP^*; R26*^DTA/DTA^) (*53*, *54*), we confirmed efficient deletion of IL-5^+^ lymphocytes, predominantly ILC2s, both at rest and after fibrosis (~80% deletion; Fig. 5, A to C). Notably, the Il5Cre allele disrupts the endogenous *Il5* locus, and both deleter mice and littermate controls similarly lack functional IL-5 with expected eosinophilopenia. Unexpectedly, constitutive loss of IL-5^+^ T2Ls resulted in elevated CCl_4_ liver fibrosis, including increased liver hydroxyproline, profibrotic gene expression, periportal ductal expansion, collagen tract deposition, and CD34 expression, a marker of periportal liver fibroblasts (*41*, *55*), whereas acute liver damage (serum ALT) was not different between groups (Fig. 5, D to J, and fig. S4A). T2L-deleter mice had decreased pericentral hepatocyte markers (Cyp2E1 and GS) (fig. S4, B and C), consistent with more severe CCl_4_-induced pericentral hepatocyte loss. These results were not secondary to early life impacts of T2L loss, as they were recapitulated in a model, allowing for inducible depletion of IL-5^+^ T2Ls in adult mice (Il5Cre^DTR^), with efficient and specific deletion (Fig. 5K and fig. S4, D to F). Fibrosis was also elevated (hydroxyproline), whereas weight loss and relative T cells and NK cells did not change (Fig. 5L and fig. S4, G to I). To compare these results with a model of cholestasis-induced liver injury, we next performed BDL surgery. IL-5–deleter mice were similar in survival, weight loss, and markers of liver injury (serum ALT and bilirubin) (Fig. 5M and fig. S4, J to M); however, they had enhanced fibrosis, with increased hydroxyproline levels, Sirius red and trichrome staining, and Col1 staining (Fig. 5, N to Q). Together, these findings suggest that the loss of IL-5^+^ T2Ls unexpectedly worsens liver fibrosis in both CCl_4_ and BDL mouse models.

### Neither IL-33 nor IL-4/IL-13 signals functionally affect CCL_4_- or BDL-driven hepatic fibrosis

Given that fibrosis induced by CCl_4_ treatment and BDL surgery was exacerbated by the loss of IL-5^+^ T2Ls, we asked whether the upstream T2L-driving cytokine IL-33 might also contribute. Although IL-33 treatment drove liver ILC2 accumulation (fig. S5, A to C), pretreatment did not alter the subsequent course of CCl_4_ injury or fibrosis, with similar levels of ALT, hydroxyproline, and several lymphocyte subsets tested (fig. S5, D to I). Next, we treated IL-33–deficient mice and heterozygous littermates with CCl_4_. IL-33–deficient mice showed no significant difference in liver fibrosis, damage markers, or relative expansion of liver ILC2s (fig. S5, J to N and R). Similarly, IL-33 deficiency did not alter BDL-induced liver disease/fibrosis indicators (fig. S5, O to R). We conclude that the loss of IL-33 does not significantly alter liver fibrosis or impair the accumulation of liver ILC2s, at least in the two mouse models tested; furthermore, transiently driving elevated liver ILC2s via IL-33 was not sufficient to alter subsequent fibrosis.

Tissue ILC2s produce constitutive IL-5 and variable IL-13 downstream of multiple activating signals (*34*, *53*, *56*), with IL-13 affecting distinct aspects of liver fibrosis, steatosis, cholestasis, and ductular reaction in type 2–driven models of liver fibrosis (*21*). To directly test the role of IL-13 in liver fibrosis in our models, we first induced IL-13 overexpression via hydrodynamic tail vein injection (*21*). This resulted in liver inflammation with increased total liver leukocytes and fibrosis (fig. S6, A to G), consistent with prior work (*21*). We recapitulated these results with mice subjected to a lower dose of IL-13 overexpression, although IL-5^+^ T2Ls were not significantly elevated by this regimen (fig. S6, H to K). These results confirmed that IL-13 excess was sufficient to drive liver inflammation and fibrosis. To address the role of endogenous IL-4/IL-13 signaling, we tested fibrosis in mice lacking the shared co-receptor component for both IL-4 and IL-13 signaling (IL-4Ra–deficient) (*57*). Following CCl_4_ treatment, there was no difference in the degree of liver fibrosis in IL4Ra^−/−^ mice relative to sex- and age-matched controls; similar to IL-33–deficient mice, liver ILC2s were not altered (fig. S6, L, M, and P). BDL-induced fibrosis yielded similar results, with no alteration in mouse survival/weight, liver damage, or fibrosis (fig. S6, N to S). These data suggest that overexpression of IL-13 can drive severe liver inflammation and fibrosis; however, neither IL-33 nor IL-4/IL-13 signaling influences the development of hepatic fibrosis from CCl_4_ or BDL surgery fibrosis, perhaps because T2Ls are not sufficiently activated to produce pathological levels of IL-13 nor sufficiently lost to trigger the associated worsened fibrosis.

### Loss of T2Ls further elevates type 3 immunity during hepatic fibrosis

**Fig. 6.**
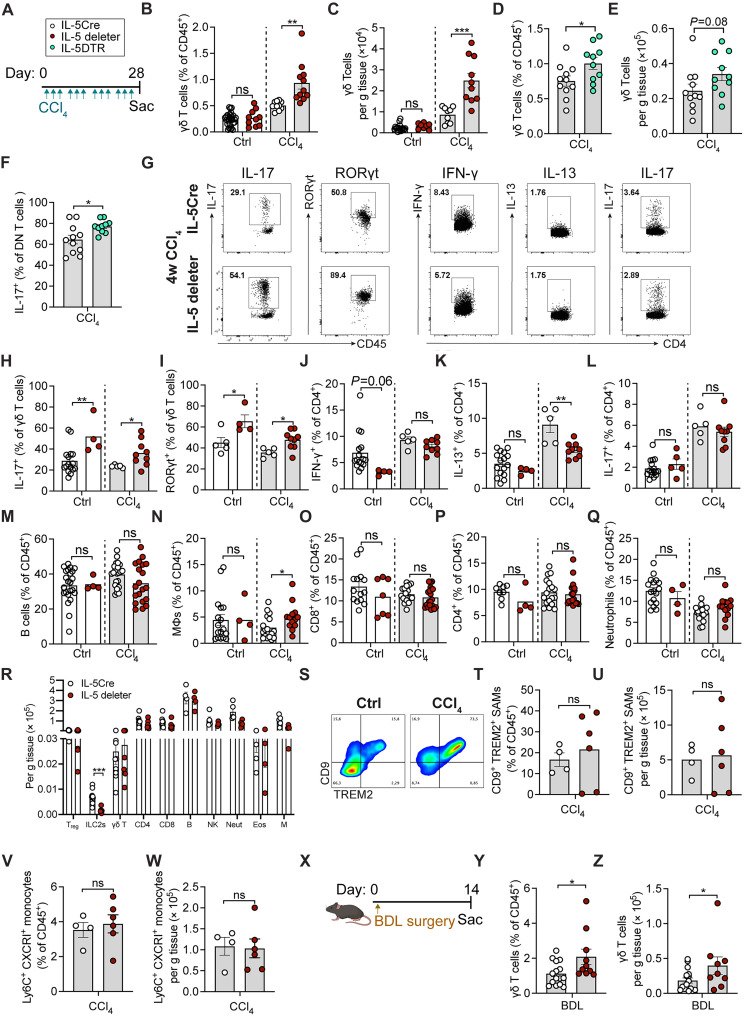
Loss of T2Ls further elevates type 3 immunity during hepatic fibrosis. (**A**) CCl_4_ schedule in Il5-tdtomato-Cre and IL-5–deleter (*Il5*^Cre-RFP/Cre-RFP^*;R26R*^DTA/DTA^) mice: 0.5 μl CCl_4_/g BW ip, three times per week for 2 or 4 weeks. (**B** and **C**) Flow quantification of percentages (B) and total numbers (C) of γδ T cells in livers from Il5-tdtomato-Cre and IL-5–deleter mice treated with vehicle or CCl_4_. Pooled data from three experiments; *n* ≥ 4 per group. (**D** to **F**) Flow quantification of percentages (D) and total numbers (E) of γδ T cells and percent IL-17^+^ DN T cells (F) in livers from Il5-tdtomato-Cre and IL-5^DTR^ (*Il5*^Cre-RFP/Cre-RFP^*;R26R*^DTR/DTR^) mice ± CCl_4_. Mice received 500 ng of DT every other day. Pooled from two experiments; *n* ≥ 5 per group. (**G** to **L**) Representative flow plots and quantitation of IL-17 [(G) and (H)] and RORγt {[(G) and (I)]; gated on CD45^+^CD19^−^CD11b^−^Thy1.2^+^NK1.1^−^CD4^−^CD8^−^]} in hepatic γδ T cells and IFN-γ [(G) and (J)], IL-13 [(G) and (K)], and IL-17 {[(G) and (L)]; gated on CD45^+^CD19^−^CD11b^−^Thy1.2^+^NK1.1^−^CD4^+^]} in hepatic CD4^+^ T cells. *n* ≥ 5 per group. (**M** to **Q**) Flow quantification of percent B cells (M), macrophages (N), CD8^+^ T cells (O), CD4^+^ T cells (P), and neutrophils (Q) in livers ± CCl_4_. Pooled from three experiments; *n* ≥ 4 mice per group. (**R**) Total numbers of indicated cells at the steady state. *n* ≥ 5 per group. (**S**) Representative flow plots of *CD9^+^TREM*^+^ SAMs in livers from IL-5tdtomato-Cre mice ± CCl_4_ (4 weeks). (**T** to **W**) Percentages [(T) and (V)] and total numbers [(U) and (W)] of *CD9^+^TREM^+^* SAMs and *Ly6C^+^CX3CRI*^+^ monocytes in livers ± CCl_4_. Pooled from two experiments; *n* ≥ 2 per group. (**X**) BDL schedule. (**Y** and **Z**) Percentages (Y) and total numbers (Z) of γδ T cells 14 days post-BDL. Pooled from three experiments; *n* ≥ 3 per group. Bar graphs indicate the means (±SE). Student’s *t* test [(B) to (F), (H) to (R), (T) to (W), (Y), and (Z)]. *P ≤ 0.05, **P ≤ 0.01, and ***P ≤ 0.001.

To better define how the loss of T2Ls was leading to more severe liver fibrosis, we next examined the liver fibrosis–associated immune landscape. During CCl_4_-induced fibrosis, T2L deficiency was accompanied by a further increase in γδ T cells; these results were largely recapitulated with inducible IL-5^+^ T2L depletion (Il5Cre^DTR^) (Fig. 6, A to E). Moreover, γδ T cells displayed an expanded type 3/17 skewing, with increased RORγt and IL-17A production (Fig. 6, F to I). Notably, even under resting conditions, IL-5–deleter mice displayed a higher percentage of IL-17^+^ and RORγt^+^ γδ T cells (Fig. 6, H and I). In contrast, while the loss of IL-5^+^ T2Ls reduced IL-13^+^ lymphocytes, as expected, it did not consistently alter T_H_1 cells (IFN-γ^+^), T_H_17 cells (IL-17A^+^), B cells, macrophages, CD8^+^ T cells, CD4^+^ T cells, or neutrophils in fibrotic livers (Fig. 6, G and J to Q). ILC2s were significantly and specifically decreased in resting IL-5–deleter mice, whereas total numbers of T_reg_ cells, γδ T cells, CD4^+^ and CD8^+^ T cells, B cells, NK cells, neutrophils, eosinophils, and macrophages were minimally affected (Fig. 6R). Previously described *CD9^+^TREM2^+^* injury-associated macrophages (*58*, *59*) expanded in CCl_4_-induced liver fibrosis but did not significantly further expand with the loss of T2Ls (Fig. 6, S to U), while *Ly6C^+^CXCR1^+^* monocytes were not altered in IL-5–deleter mice (Fig. 6, V and W). After BDL surgery, γδ T cells were also further increased in T2L-deleter mice (Fig. 6, X to Z). These data suggest a critical and specific role of IL-5^+^ T2Ls in restraining T3Ls and type 3/17 immunity during hepatic fibrosis.

### Redundancy in T3Ls during hepatic fibrosis

We next tested whether mice deficient in γδ T cells (*TCRd*^−/−^) would be protected against CCl_4_-driven liver fibrosis (fig. S7, A to C). T*CRd*^−/−^ mice had similar degrees of liver fibrosis and ILC2 expansion (fig. S7, D to F), suggesting that the loss of γδ T cells alone was not sufficient to alter CCl_4_-induced fibrosis. However, levels of other IL-17A–producing innate-like T cells were notably increased in CCl_4_-treated T*CRd*^−/−^ mice (fig. S7G), indicating potential T3L compensation in the absence of γδ T cells, possibly via MAIT cells or ILC3s (*60*). RORγt regulates the differentiation of type 3 immune cells and IL-17A production (*61*, *62*), and antagonizing RORγt transcriptional activity could limit excessive IL-17 responses associated with the loss of IL-5^+^ lymphocytes. To test this, we treated IL-5–deleter mice daily with a RORγt antagonist (GSK 805), starting after 2 weeks of CCl_4_ treatment (fig. S7H). Liver damage or fibrosis was not reduced, although T_H_17 frequencies were mildly decreased (fig. S7, I to L). γδ T cells and IL-17A^+^ innate-like T cells were increased (fig. S7, M and N), again suggesting compensation between T3L subsets. To directly test the role of the IL-17A cytokine itself, we administered an anti–IL-17A neutralizing antibody; neutrophil frequencies were decreased in anti–IL-17A–treated IL-5–deleter mice, but IL-17–producing γδ T cells were further elevated, and liver damage and fibrosis markers were minimally altered (fig. S7, O to S). These data indicate redundancy in T3Ls and their signals that may mediate liver fibrosis, at least in the context of T2L deficiency.

**Fig. 7.**
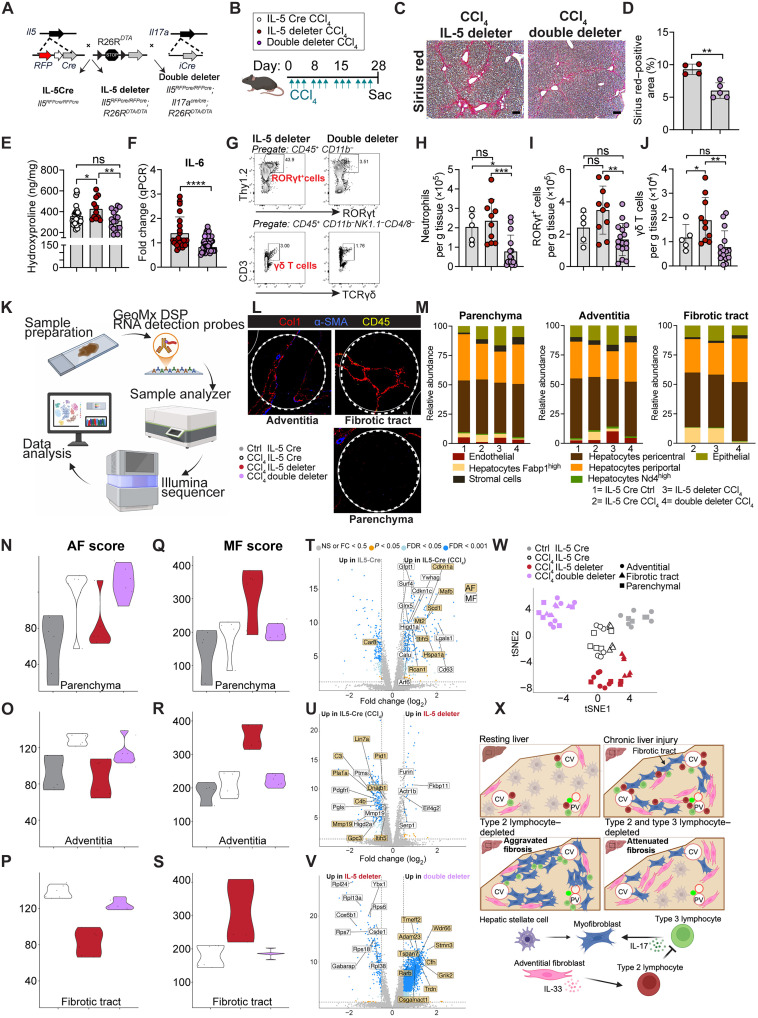
Depletion of IL-17 reduces advanced hepatic fibrosis. (**A**) Schematic of IL-17; IL-5 double-deleter mice (Il17aCre; *Il5*^Cre-RFP/Cre-RFP^*;R26R*^DTA/DTA^) and IL-5 deleter controls (*Il5*^Cre-RFP/Cre-RFP^*;R26R*^DTA/DTA^). (**B**) CCl_4_ treatment schedule: 0.5 μl CCl_4_/g BW ip, three times per week for 4 weeks. (**C** and **D**) Sirius red staining of 5-μm liver sections (C) and quantification (D) after 4-week CCl_4_ treatment. Pooled from two experiments; *n* ≥ 4 per group. Scale bar, 200µm. (**E**) Hepatic hydroxyproline quantification; pooled from two experiments; *n* ≥ 5 per group. (**F**) Liver IL-6 expression normalized to 36B4 in 4-week CCl_4_–treated IL-5–deleter mice and double-deleter mice. *n* ≥ 8 per group, two experiments. (**G**) Flow plots showing deletion of RORγt^+^ cells (upper) and γδ T cells (lower) in double-deleter versus IL-5–deleter mice. (**H** to **J**) Flow quantification of neutrophils (H), RORγt cells (I), and γδ T cells (J) in livers of Il5-tdtomato-Cre, IL-5–deleter, and double-deleter mice after 4-week CCl_4_ treatment. Pooled from two experiments; *n* ≥ 3 per group. (**K**) GeoMx workflow. Drawn with BioRender. J. Sbierski-Kind (2025); https://biorender.com/f42j576. (**L**) Representative staining for Col1 (fibrotic tract), α-SMA (vessels), CD45 (leukocytes) in IL-5–deleter mice, with indicated ROIs for GeoMx. (**M**) Cell abundances for parenchymal, adventitial, and fibrotic tract areas estimated using SpatialDecon. (**N** to **S**) AF (525 genes) [(N) to (P)] and MF [521 genes (*65*)] scores [(Q) to (S)] in vehicle-treated Il5-tdtomato-Cre mice (gray), CCl_4_-treated Il5-tdtomato-Cre mice (white), IL-5–deleter (red), and double-deleter (purple) mice. (**T** to **V**) Volcano plots of top 10 differentially expressed genes for AF and MF scores (*70*): CCl_4_-treated Il5-tdtomato-Cre versus vehicle (T), IL-5–deleter versus Il5-tdtomato-Cre mice (U), and double-deleter versus IL-5–deleter mice (V). (**W**) tSNE of fibrotic tract, adventitial, and parenchymal areas under indicated conditions. (**X**) Graphic scheme. Created in BioRender [J. Sbierski-Kind (2025); https://biorender.com/mx6h6dl]. Bar graphs indicate the means (±SE). Student’s *t* test [(D) and (F)] and one-way ANOVA with Tukey post test [(E) and (H) to (J)]. *P ≤ 0.05, **P ≤ 0.01, and ***P ≤ 0.001. See also fig. S7.

To overcome this limitation and potential feedback mechanisms, we next used a genetic approach to concurrently reduce all IL-17A^+^ T3Ls along with IL-5^+^ T2Ls (IL-17A^Cre^; *Il5*^Cre-RFP/Cre-RFP^*;R26*^DTA/DTA^) (*51*), hereafter referred to as double-deleter mice (Fig. 7A). We subjected double-deleter mice, and their IL-5–deleter controls, to 4 weeks of CCl_4_ treatment (Fig. 7B). Liver fibrosis and inflammation markers were reduced in double-deleter mice as compared to IL-5–deleter mice (Fig. 7, C to F), as were total liver neutrophils, RORγt^+^ cells, and γδ T cells (Fig. 7, G to J).

### Spatial heterogeneity of the stromal and immune landscape upon the loss of T2Ls and T3Ls

To gain insight into the cellular composition and functional relationships within distinct liver niches in mice deficient in IL-5^+^ T2Ls and/or IL-17A^+^ T3Ls, we performed spatial transcriptomics using the GeoMx whole-transcriptome platform (*63*) and defined regions within healthy and fibrotic liver tissues that were enriched for parenchyma, adventitia, or fibrotic tracts (Fig. 7, K and L). Consistent with our microscopic analysis, double-deleter mice showed a transcriptomic pattern consistent with less severe CCl_4_-induced pericentral hepatocyte loss and fibrosis and decreased frequencies of epithelial (duct) cells and disease-progressing Fabp1^high^ hepatocytes (Fig. 7M), as inferred from the ImmGen dataset used for deconvolution (*64*). In contrast, T2L deficiency resulted in the enhanced relative abundance of endothelial, stromal, and epithelial cell signatures (Fig. 7M), concomitant with duct and stromal cell proliferation visualized via microscopy.

To study the impact of both losses of T2Ls and T3Ls on AFs and MFs in the context of hepatic fibrosis, we developed a multigene AF-associated transcriptomic score, representing genes elevated in AFs, and an MF-associated score, calculated by using all differentially expressed markers of Lrrc15^+^ MFs from Buechler *et al.* (*65*). We found elevated scores for both AFs and MFs within CCl_4_-treated control mice (IL-5Cre) across regions tested (Fig. 7, N to S). Simultaneously, both a pan-fibroblast score (*46*) and periportal hepatocyte score were increased with liver fibrosis, whereas a pericentral hepatocyte score (*66*) declined (fig. S7, T to V). In addition, the pan-fibroblast signature was elevated in CCl_4_-treated IL-5 Cre mice (fig. S7W). Consistent with the exacerbation of fibrosis, IL-5 T2L–deleter mice treated with CCl_4_ exhibited a reduced AF score (Fig. 7, N to P), accompanied by a further elevated MF score (Fig. 7, Q to S). In contrast, the AF score was increased in double-deleter mice (Fig. 7, N to P), while the MF score showed a corresponding decrease (Fig. 7, Q to S). Next, we compared the top 10 differentially expressed genes from AF and MF scores in CCl_4_-treated double-deleter mice, IL-5–deleter mice, and control and CCl_4_-treated IL-5Cre mice (Fig. 7, T to V). Both AF and MF signature genes increased with fibrosis (Fig. 7T), whereas AF and MF scores displayed opposing regulation in IL-5–deleter and double-deleter mice (Fig. 7, U and V). t-Distributed stochastic neighbor embedding (tSNE) representation highlighted distinct clustering among each of the different genotypic groups (Fig. 7W). The scar-associated macrophage (SAM) score, derived from SAM signature genes described by Fabre *et al.* (*58*), was increased with liver injury–induced fibrosis without clear alterations across genotypes (fig. S7X), consistent with our flow cytometry results (Fig. 6, S to U). Together, our findings suggest a model in which T2Ls and T3Ls share resting and expanded postfibrotic liver niches, defined at least in part by niche AF-like fibroblasts, and the loss of T2Ls leads to aggravated T3L-driven inflammation, a further expansion of MFs within these shared fibrotic regions, and ultimately, aggravated liver fibrosis (Fig. 7X).

## DISCUSSION

Fibrosis is often driven by dysregulated repair during repetitive tissue injuries. Although lymphocytes and their cytokines influence tissue fibrosis, the precise spatial contributions are poorly defined. We previously identified adventitial niches around larger vessels and other tissue border sites, where AFs marked by high IL-33 levels locally regulate T2Ls. Additional immune and nonimmune subsets are also enriched at these regional immune hubs (*35*, *67*, *68*). AFs across multiple tissues share a universal fibroblast signature with increased mesenchymal progenitor capacity, suggesting a potential broadly conserved immunomodulatory role (*65*). Here, we found that chronic liver injury and fibrosis caused the expansion of fibroblasts. Unexpectedly, these fibroblasts were not only traditional profibrotic MFs but AF-like niche fibroblasts that were particularly associated with expanded T2Ls and T3Ls. Niche fibroblasts were IL-33^high^ and were distinct from MFs that expressed α-SMA^+^ (*Acta2* gene) and TGF-β–driven Cthrc1, although they existed in proximity within collagen-dense fibrotic areas. Immune cells were broadly associated with these fibrotic regions, and fibrosis concurrently expanded both T2Ls and T3Ls. The loss of T2Ls further unleashed liver fibroblast–associated T3L immune accumulation, cytokine response, relative expansion of MFs, and fibrosis across two commonly used mouse models of liver fibrosis.

Chronic and inappropriate activation of type 2 immunity drives allergic diseases and can synergize with fibrotic signals like TGF-β to promote pathological fibrosis. In type 2 immune-skewed liver fibrosis (e.g., *Schistosoma* egg deposition), IL-13 drives hepatic fibrosis, steatosis, cholestasis, and ductular reaction, simultaneously acting on distinct cellular targets (*21*). Excess IL-33, IL-25, and TSLP, all tissue cytokines that drive type 2 immunity, can each lead to organ fibrosis (*24*, *29*, *69*), with combinatorial targeting of all three signals reducing type 2–driven inflammation and fibrosis in infectious parasitic models (*53*, *70*). However, we found that limited exposure to IL-33, with concomitant ILC2 expansion, was insufficient to alter CCl_4_-driven liver fibrosis; furthermore, although we confirmed that IL-13 excess can drive liver fibrosis, lack of IL-4Rα (and associated IL-4/IL-13 signaling) did not alter liver fibrosis in CCl_4_ models. Type 2 immune activation can represent an adaptive response to tissue perturbation, promoting the resolution of inflammation and protecting against excessive lung injury (*53*, *71*–*73*). One parsimonious conclusion is that while excessive type 2 immunity is sufficient to promote fibrotic disease, its role in vivo is context-, timing-, and disease-dependent.

Elevated T3Ls and associated IL-17A and/or IL-22 cytokines have emerged as drivers of fibrosis in chronic or repetitive liver injuries (*17*, *74*). IL-17A has profibrogenic functions, promoting increased production of TGF-β (*75*), recruitment of pro-inflammatory neutrophils and monocytes, and activation of the STAT3 (signal transducer and activator of transcription 3) pathway that induces Col1 production in liver stromal cells (*74*). IL-17A can also enhance the sensitivity of fibroblasts to TGF-β by increasing the expression of TGF-βRII (*16*). Recent work suggests that tissue-resident liver innate-like T cells secrete IL-17A, are activated through NKG2D, and license hepatocytes to produce chemokines that recruit pro-inflammatory cells into the liver to worsen fibrosis (*76*). These observations are consistent with our findings that IL-17A^+^ lymphocytes accumulated in collagen-dense fibrous bands and correlated with MF accumulation and fibrosis severity; furthermore, T3L loss attenuated the excessive hepatic fibrosis observed with the loss of T2Ls. Targeting the type 3 immune axis may represent a viable strategy in various liver diseases (*76*, *77*). In our study, IL-5–deleter mice showed a marked accumulation of IL-17^+^ γδ T cells and aggravated fibrosis, underscoring the fact that IL-17 signaling is active in this setting. Our observed lack of effect of antibody-mediated IL-17 blockade may reflect the redundancy of IL-17 biology, including possible compensation by IL-17F or IL-17A/F heterodimers, incomplete neutralization of IL-17A at the site of injury, or IL-17–independent profibrotic activities of T3Ls.

Macrophages intimately coordinate tissue responses to injury and contribute to both fibrosis progression and resolution (*52*, *78*, *79*). Both resident and recruited macrophages localize to distinct liver zones in contact with liver sinusoidal endothelium and hepatic stellate cells. Profibrotic SAMs (CD9^+^ TREM2^+^) were enriched in the fibrotic niche adjacent to activated stromal cells, suggesting that distinct macrophage states may be an additional critical fibrotic niche component (*58*). Moreover, commensal bacterium–derived signals also organize liver immune spatial polarization, with Kupffer cells and NKT cells enriched in parenchymal zones closer to portal areas, promoting host defense (*80*). It will be interesting to investigate how the microbiome influences the liver immune zonation observed here.

Our results show a shared stromal liver niche, where T2Ls and T3Ls are in close proximity, with possible similar niches in other tissues. In contrast, T1Ls were broadly distributed across domains both at rest and in the fibrotic liver, consistent with prior work (*31*). T2Ls and T3Ls accumulated in periportal regions and fibrous bands after CCl_4_- or BDL-induced liver injury, but they did not significantly accumulate in liver parenchymal sites, a region with abundant T1Ls; nonetheless, it is possible that subsets of T1Ls are topographically similar to ILC2s and γδ T cells and could locally affect this fibroblast-immune cross-talk. We also found that the loss of IL-5^+^ T2Ls unleashed type 3–associated responses to chronic liver injury and fibrosis. Similar results were previously seen in chitin-induced type 2 lung inflammation (*53*), and type 2–like T_reg_ cells suppress innate IL-17A^+^ γδ T cell responses to mucosal injury induced by environmental allergens (*81*) and in mouse models of neuroinflammation (*82*). The cross-regulatory activity between type 2 and type 3/17 flavors of cell-mediated effector immunity is well recognized, but the understanding of the complex cell-extrinsic niches that may regulate lymphocyte residence and accumulation is only emerging (*20*). While AFs (IL-33^high^) are sufficient to support both ILC2 and γδ T cell survival, we did not observe any impact of IL-33 per se. Early-life ILC2s are also intimately associated with AFs, which produce both TSLP and IL-33, but neither signal was required for ILC2 tissue colonization or identity (*83*).

We found that both healthy and fibrotic livers contained a subset of fibroblasts that expressed high IL-33 and were enriched in periportal regions and around fibrous bands. Similarly, AFs that are primed to support type 2 immune responses (*36*) were recently referred to as “border fibroblasts” because they are located at the adventitial domain surrounding larger vessels, fascial planes, and body-cavity linings and thus help maintain tissue zonation (*84*). In contrast, MFs showed only weak or absent expression of IL-33. Singe-cell RNA sequencing of healthy and fibrotic mouse livers has revealed spatial zonation of pericyte-like HSCs (*41*). Our work does not resolve the possible lineage relationships between HSC, AF, and fibrotic tract fibroblast states. Nonetheless, our findings suggest that discrete fibroblast states are strongly associated with type 2 and 3 immune topography, and in vitro, similar niche AFs are sufficient to support both ILC2s (*32*) and γδ T cells without exogenous cytokine support or TCR activation. Consistently, depletion of T2Ls led to a reduction of AF-associated genes, whereas the MF signature was increased, as revealed by spatial transcriptomics, suggesting that T2L feedback affects fibroblast states.

Future work will be required to elucidate the likely cross-regulatory signals between T2Ls, T3Ls, stromal cell subsets, and other potential niche occupants such as dendritic cells, macrophage subsets, and lymphatics. Further exploration of the healthy and fibrotic hepatic immune-stromal interactions may delineate topographically restricted mechanisms of cross-talk and lead to disease-relevant therapeutic strategies.

### Limitations of the study

(i) IL-5–deleter mice lack T2Ls, predominantly ILC2s, in all tissues from early life; therefore, this genetic model is not tissue- or timing-specific. In addition, diphtheria toxin A (DTA)–based approaches can have unintended toxic effects beyond the targeted cell population, raising the possibility of off-target consequences. However, we were able to phenocopy critical findings in an inducible genetic depletion model for ILC2s, suggesting that functional impacts are not secondary to developmental alterations. (ii) IL-5 deficiency in IL-5–deleter mice leads to a reduction of eosinophils across tissues. Although ΔdblGATA mice (which lack eosinophils) show impaired liver regeneration after acute injury, particularly in CCl_4_ or ischemia-reperfusion models (*85*, *86*), there is, to our knowledge, little direct data in which eosinophil-deficient mice have been challenged in chronic fibrosis models such as repeated CCl_4_ treatment or BDL to assess the extent of fibrosis, stellate cell activation, or recovery. While eosinophils can contribute to tissue repair and resolution in acute injury (e.g., via IL-4/heparin‐binding epidermal growth factor–like growth factor cross-talk) (*87*), whether their absence exacerbates or mitigates fibrosis in prolonged injury remains untested and represents a gap in the literature. Although we used IL-5–deficient mice as control groups for our studies, which have profoundly reduced tissue eosinophils, our results may minimize additional impacts of eosinophils on hepatic fibrosis. (iii) This work does not dissect how γδ T cells, or other similar IL-17A^+^ T3Ls, contribute mechanistically to centrilobular liver fibrosis or portal fibrosis. Although we found that genetic ablation of IL-17A–producing cells reduced the degree of CCl_4_-induced liver fibrosis, in line with previous studies (*16*, *74*, *88*), we did not identify a unique contribution of individual cytokines (e.g., IL-17A and IL-22) or T3L subsets (e.g., γδ T cells, CD4^+^ T_H_17 cells, and subsets of MAIT or NKT cells), likely due to considerable redundancy of these cells and pathways. (iv) We did not resolve the detailed mechanistic pathways by which T2L-T3L cross-talk limits MF formation. On the basis of our findings, we propose two potential mechanisms that may both contribute to the observed T2L-T3L cross-regulation: (i) T2Ls may directly limit T3L activation, migration, and/or expansion through cell-cell contact or cytokine-mediated interactions, and (ii) the physical proximity of these subsets may allow T2Ls to indirectly regulate T3L numbers and activity via competition for fibroblast- and/or macrophage-derived growth factors.

## MATERIALS AND METHODS

### Study design

The objective of this study was to investigate the cross-talk of ILC2s with stromal cells during the development of hepatic fibrosis. We used flow cytometry, thick section confocal imaging, and different experimental models (BDL and CCl_4_) of liver fibrosis to localize and define the role of ILC2s as critical mediators of inflammation. Sample sizes that would yield 80% power to detect a 30% difference between experimental groups at a 5% significance level were determined by power calculations from pilot studies on previously published data. All experiments used randomly assigned mice without investigator blinding. Data were pooled from multiple experiments, unless otherwise specified. All data points and *n* values reflect biological replicates. For imaging experiments, at least three mice were analyzed from at least two independent experiments, with two or more fields analyzed per mouse. No data were excluded.

### Mice

Mice were bred and maintained in specific pathogen–free conditions at the animal facilities of University of California San Francisco (UCSF) and were used in accordance with institutional guidelines and under study protocols approved by the UCSF Institutional Animal Care and Use Committee (protocols AN193180-01J and AN195761-01B). All mice were 6 to 12 weeks old, unless otherwise noted. Experiments were performed with mixed sex mice backcrossed on C57BL/6 for at least 10 generations. All experimental groups were sex balanced, with equal representation of male and female mice. Red5 (Il5-tdtomato-cre) cytokine reporter mice were used for tracking IL-5–producing T2Ls (the Jackson Laboratory, 030926) (*34*). Imaging was performed in Red5 mice crossed to R26-CAG-RFP mice (Ai14) containing a flox-stop-flox sequence upstream of a CAG-RFP-WPRE-cassette in the constitutively expressed ROSA26 (R26) locus (the Jackson Laboratory, 007914) or in Red5 mice crossed to R26-CAG-YFP mice (Ai3) (*89*), serving as IL-5 lineage reporters (*32*). To delete ILC2s, Red5 (Il5-tdtomato-Cre) mice were intercrossed with R26-DTA, an approach that specifically deletes ~70 to 80% of ILC2s, as described previously (*34*). To conditionally delete ILC2s, Red5 (Il5-tdtomato-Cre) mice were intercrossed with R26-DTR mice, generously provided by A. V. Molofsky and previously described (*90*). To concurrently delete IL-17A^+^ T3Ls and IL-5^+^ T2Ls, we used IL-17ACre mice (*51*) crossed with IL-5–deleter mice (*Il5*^Cre-RFP/Cre-RFP^*;R26R*^DTA/DTA^). Additional mice used include genetically targeted IL-33^mcherry^ (*91*), PDGFRα-H2B-eGFP nuclear-localized GFP (the Jackson Laboratory, 007669), RORγt-GFP (*50*), *Arg1*^Yarg^ [Yarg; B6.129S4-*Arg1^tm1Lky^*/J; 015857; (*92*)], *Il13*^Smart^ [Smart13; B6.129S4[C]-*Il13^tm2.1Lky^*/J; 031367; (*93*)], and *Arg1*^RFP-CreERT2^ (*94*) mice. PDGFRα-H2B-eGFP; FoxP3DTR/α-SMA-RFP mice were generated by crossing PDGFRα-H2B-eGFP to FoxP3DTR/α-SMA-RFP mice, which were provided by M. Rosenblum and were previously described (*95*). Tbet (Tbx21)-ZsGreen transgenic mice were a gift of J. Zhu, Lab of Immune System Biology, National Institutes of Health (NIH), and were previously described (*96*). IL4ra^−/−^ mice were generated as previously described (*57*) and were provided by F. Brombacher. *TCRd*^−/−^ (the Jackson Laboratory, 002120) and C57BL/6J (the Jackson Laboratory, 000664) mice were purchased from the Jackson Laboratory (*95*). Acta2^creERT2^ mice were used to track MFs and smooth muscle cells, and Cthrc2^creER^ mice (provided by D. Sheppard) were used to track MFs (*38*). To lineage trace resting and liver injury–responsive fibroblasts, we used Col1a2creERT2; Rosa26^Tdt-Ai14^ mice, as previously described (*39*). To track AFs, we used Rosa26^Tdt-Ai14^ mice crossed to Gli1^creERT2^ mice (the Jackson Laboratory, 007913) (*32*).

### CCl_4_ treatment

Mice were treated with CCl_4_ (Sigma-Aldrich, cat. no. 289116), resuspended in corn oil, at 0.5 ml/kg or vehicle (corn oil) with three intraperitoneal injections per week for the indicated duration.

### Bile duct ligation

All procedures were carried out under clean but nonsterile conditions following standard operating aseptic protocols. Mice were anesthetized with inhalation of isoflurane after intraperitoneal injection of ketamine/xylazine [ketamine (80 to 100 mg/kg) + xylazine (5 to 10 mg/kg)] and buprenorphine analgesia (0.05 to 0.1 mg/kg) administered via subcutaneous injection. The abdomen was opened by a midline laparotomy ~2 cm in length with a surgical scissor, and the peritoneum was cut open along the linea alba. The peritoneal cavity was enlarged by inserting a Colibri retractor, and the hilum was revealed using a moisturized (0.9% NaCl solution) cotton swab. The bile duct was exposed and separated from the PV and hepatic artery using a microserration forceps. The 7-0 suture was placed around the bile duct and secured with two surgical knots. A second cranial ligation was added in the same manner. Abdominal layers (peritoneum and cutis plus facia) were closed with running 6-0 sutures with absorbable suture material. Mice were allowed to recover in a cage warmed up by an infrared lamp until they were fully awake and active. Mice were weighed daily and monitored for humane end point. Mice were euthanized after either 7 or 14 days.

### Administration of DT

DT (Sigma-Aldrich) was administered intraperitoneally at a dose of 500 ng in sterile phosphate-buffered saline (PBS) initially 2 or 1 day before CCl_4_ injections and then every other day throughout the experiment.

### Cytokines, neutralizing antibodies, and chemicals

For cytokine injections, IL-33 (BioLegend) was given intraperitoneally as 500 ng in 0.2 ml of PBS every day for three doses. For the therapeutic intervention, mice were placed on CCl_4_ [0.5 ml/kg, intraperitoneally (ip), three times per week] for 4 weeks and treated with 250 μg of mouse anti–IL-17a (clone 17F3, BioXcell) antibody intraperitoneally in a volume of 200 μl (diluted in PBS) at the beginning of CCl_4_ application and throughout the experiment or with the RORγt antagonist GSK805 (Sigma-Aldrich, cat. no. 5313690001) from weeks 2 to 4 daily at 10 mg/kg ip in corn oil. Vehicle (corn oil) was used as a control. For bleomycin injury, mice were given pharmaceutical-grade bleomycin (Hospira) dissolved in PBS via intranasal application once a week for 4 weeks, as previously described (*97*). Mice were given a dose of 0.75 U/kg per dose.

### Hydrodynamic delivery of IL-13

Mice were injected intravenously with 1 or 10 μg of a mammalian expression plasmid coding for murine IL-13 in 1 ml of warm saline. Control mice were injected with warm saline (*98*).

### Flow cytometry

Single-cell suspensions were prepared from tissues including blood, liver, lung, and epigonadal white adipose tissue. After euthanizing the mice with CO_2_, peripheral blood was collected through cardiac punction, and mice were perfused with 10 ml of PBS via the left ventricle. Livers were weighed, cut in small pieces with an automated tissue dissociator (Gentle Macs; Miltenyi Biotec), and then digested in 7 ml of Hank’s balanced salt solution with 0.5% bovine serum albumin (Sigma-Aldrich, cat. no. A2153), 2% fetal bovine serum (FBS), 40 μl of Liberase (0.2 mg/ml, Roche, cat. no. 5401127001), and 40 μl of deoxyribonuclease I (DNase I; 10 mg/ml, Roche, cat. no. 10104159001) for 30 min at 37°C with gentle agitation. Samples were subsequently processed on the GentleMacs using the “lung2” program, passed through 70-μm filters, and centrifuged at 30*g* for 3 min at 4°C to remove hepatocytes. The supernatant was centrifuged, and leukocytes were further separated using 40% Percoll density gradient (GE Healthcare, cat. no. 17-0891-01) and centrifugation (1400*g*, 20 min, room temperature, no brake), followed by red blood cell lysis (PharmLyse; BS Biosciences) before final suspension in fluorescence-activated cell sorting (FACS) buffer (PBS, 3% FBS, and 0.05% NaN_3_). Whole-lung single-cell suspensions were prepared as previously described (*32*) by harvesting lung lobes into 5 ml of Hank’s balanced salt solution with 40 μl of Liberase (0.1 wU/ml, Roche, cat. no. 5401127001) and 20 μl of DNase I (10 mg/ml, Roche, cat. no. 10104159001), followed by automated tissue dissociation (GentleMacs; Miltenyi Biotec) and tissue digestion for 30 min at 37°C on a shaker. Digested samples were processed on the GentleMacs using the “lung2” program, passed through 70-μm filters, and washed, followed by red blood cell lysis and final suspension in FACS buffer. The epigonadal white adipose tissue was harvested; cut into small pieces with a scissor; digested in 10 ml of low-glucose Dulbecco’s modified Eagle’s medium (DMEM) containing Liberase (0.2 mg/ml), DNase (25 μg/ml), 0.2 M Hepes, and bovine serum albumin (BSA; 10 mg/ml) for 45 min at 37°C with gentle agitation; passed through 100-μm filters; and centrifuged at 1000*g* for 10 min. Red blood cells were lysed with PharmLyse, and the remaining cell pellets were resuspended in FACS buffer. Blood samples were centrifuged for 5 min at 1500*g* and resuspended in PharmLyse for ~10 min at room temperature, followed by centrifugation and final suspension in FACS buffer. Cells were counted using a NucleoCounter (Chemometic). All samples were stained in 96-well V-bottom plates. Single-cell samples were first incubated with antibodies to surface antigens for 30 min at 4°C in a 50-μl staining volume. For intracellular staining, cells were fixed and permeabilized using the FoxP3/Transcription Factor Staining Buffer Set (eBioscience, cat. no. 00-5523-00). For cytokine staining, 3 × 10^6^ cells were plated in 96-well U-bottom plates and stimulated ex vivo with phorbol 12-myristate 13-acetate (30 ng/ml; Sigma-Aldrich, cat. no. 79346-1mG) and ionomycin (500 ng/ml; Sigma-Aldrich, cat. no. IO634-5MG) in culture medium [RPMI 1640 + 10% FBS, 10% penicillin/streptomycin, 50 mM 2-mercaptoethanol (Sigma-Aldrich, cat. no. 60-24-2), and 1000× BD Golgi Plug containing Brefeldin A (Sigma-Aldrich, cat. no. 555029)] for 3 hours at 37°C. Flow cytometry was performed on a BD LSRFortessa X-20. Fluorochrome compensation was performed with single-stained UltraComp eBeads (Invitrogen, cat. no. 01-2222-42). Samples were FSC-A (forward scatter area)/SSC-A (side scatter area) gated to exclude debris, followed by FSC-H (forward scatter height)/FSC-A gating to select single cells and Zombie NIR fixable or DAPI (4′,6-diamidino-2-phenylindole) to exclude dead cells. ILC2s were identified as lineage-negative (CD11b^−^, CD3ε^−^, CD4^−^, CD8α^−^, CD19^−^, NK1.1^−^), CD45^+^, Thy1.2 (CD90.2^+^), and Gata3^hi^, IL1RL1 (ST2^+^), or CD25 (IL-2Rα^+^), as indicated. T_H_2 cells were identified as CD45^+^, CD3ε^+^, CD4^+^, and FoxP3^−^. For some analyses, IL-5 lineage tracker mice (Il5-tdtomato-Cre; R26-RFP) were used to gate ILC2s as lineage-negative, Thy1.2 (CD90.2^+^), RFP^+^ and T_H_2 cells as CD45^+^, CD3ε^+^, CD4^+^, RFP^+^. Data were analyzed using FlowJo software (TreeStar, US) and compiled using Prism (GraphPad software).

### Flow cytometry antibodies

Monoclonal antibodies used for flow cytometry include anti-CD45 (30-F11, BioLegend), anti-CD90.2 (Thy1.2) (53-2.1, BioLegend), anti-CD3 (17A2, BioLegend), anti-CD4 (RM4-5, BioLegend), anti-CD8 (53-6.7 BioLegend), anti-CD11b (M1/70, BioLegend), anti-CD11c (N418, BioLegend), anti-NK1.1 (PK136, BioLegend), anti-CD19 (6D5, BioLegend), anti–TCRγ/δ (GL3, BioLegend), anti–T1/ST2 (DJ8, MD BioSciences), anti-KLRG1 (2F1, BioLegend), anti-IL13 (eBio13A, eBioscience), anti-MerTK (DS5MMER, eBiosciences), anti-64 (X54-5/7, eBiosciences), anti-Ly6C (HK1.4, BioLegend), anti-Ly6G (RB6-8C5), anti-SiglecF (E50-2440), anti–I-A/I-E (MHCII) (M5/114.15.2, BioLegend), anti-CD25 (PC61, BioLegend), anti–IFN-γ (XMG1.2, BioLegend), anti-IL17A (TC11-18H10.1, BioLegend), and anti-RORγt (B2D, eBioscience). Anti-FoxP3 (FJK-16S, eBiosciences) and anti-GATA3 (TWAJ, eBiosciences) were used after first using a fixable live/dead stain (Invitrogen), then fixing, and permeabilizing cells per the manufacturer’s instructions. For nonhematopoietic cells, antibodies used include anti-CD31 (390, BioLegend), anti-EpCAM (CD326, G8.8, eBiosciences), anti-Gp38 (podoplanin) (8.1.1, BioLegend), anti-PDGFRα (CD140a, APA5, BioLegend), and anti-Ly6A/E (Sca1, D7, eBioscience).

### Imaging antibodies

Primary antibodies used for imaging include Living Colors anti-DsRed Rabbit Polyclonal Pan Antibody (1:500; TaKaRa), Chicken Polyclonal anti-GFP (1:300, Aves Labs), Alexa Fluor 488 anti-aSMA monoclonal Antibody (IA4, 1:200; eBioscience), eFluor 660 anti-LYVE1 monoclonal Antibody (ALY7, 1:500, eBioscience), Goat Monoclonal anti-mouse Collagen Type 1 (1:200, Southern Biotech), Rat Monoclonal anti-mouse CD326 (G8.8, 1:200, BD Pharmingen), Rabbit Monoclonal anti-mouse CD34 (EP373Y, 1:200, Abcam), Mouse Monoclonal anti-mouse Glutamine Synthetase (GS-6, 1:250, Millipore), Rabbit Polyclonal anti-SDS (PA5-58704), eBiosciences), Goat Monoclonal anti-mouse Desmin (GWB-EV0472, 1:200, Genway Biotech), Goat Polyclonal anti-VEGFR3 (R&D Systems), Rabbit Monoclonal anti-mouse Vimentin (EPR3776, 1:200, Abcam), and Rabbit Polyclonal anti-Cytochrome P450 2E1 (1:250, Abcam). The following secondary antibodies were used at 1:300 dilution: Alexa Fluor 555 donkey anti-rabbit immunoglobulin G (IgG) (H + L) cross-adsorbed (Thermo Fisher Scientific), Alexa Fluor 647 donkey anti-rat IgG (H + L) cross-adsorbed (Abcam), Alexa Fluor 647 donkey anti-rabbit IgG (H + L) cross-adsorbed (Life Technologies), Alexa Fluor 488 donkey anti-rat IgG (H + L) cross-adsorbed (Abcam), Alexa Fluor 488 donkey anti-chicken IgG (H + L) cross-adsorbed (Sigma-Aldrich), Alexa Fluor 647 donkey anti-goat IgG (H + L) cross-adsorbed (Thermo Fisher Scientific), and Alexa Fluor 555 donkey anti-mouse IgG (H + L) cross-adsorbed (Thermo Fisher Scientific).

### 3D tissue preparation and imaging

Animals were euthanized with CO_2_, followed by transcardial perfusion with 20 ml of PBS and 4% paraformaldehyde (PFA) (Thermo Fisher Scientific, cat. no. 28906). Tissues (liver, adipose tissue, gall bladder, and lung) were harvested and fixed in fresh 4% PFA overnight at 4°C. Fixed tissues were cut into ~200-μm-thick sections using a vibratome (Leica VT 1000S or Precisionart Compresstome VF-310-0Z). Tissue slices were permeabilized (PBS/0.2%Triton X-100/0.3 M glycine) and then blocked in PBS, 0.2% Triton X-100, 10% FBS, 1% BSA, and 5% serum (appropriate species serum for secondary antibodies) at 4°C overnight. Samples were then incubated with primary antibodies in PBS, 0.2% Triton X-100, and 3% serum at 4°C until the next day. Next, samples were washed three or four times in PBS and 0.2% Triton X-100 for 30 min and then incubated with secondary antibodies diluted in PBS, 0.2% Triton X-100, 10% FBS, 1% BSA, and 5% serum at 4°C overnight. Samples were then washed three or four times in PBS and 0.2% Triton X-100 for 30 min; dehydrated in an ascending ethanol series (20, 30, 50, 70, 95, and 100%), 10 min each step; and cleared in methyl salicylate. For identification of ILC2s, liver and lung tissues were stained with anti-tdtomato (1:200). For localization of ILC2s to anatomical structures, samples were stained with anti-aSMA (1:200), anti-Lyve 1 (1:500), anti–type 1 collagen (1:200), anti-CD326 (1:200), anti-CD34 (1:200), anti-Desmin (1:200), and anti-Vimentin (EPR3776, 1:200, Abcam). All samples were scanned using a Nikon A1R laser scanning confocal microscope including 405-, 488-, 561-, and 650-nm laser lines for excitation and imaged with a 16×/0.8–numerical aperture Plan Apo long working distance water immersion objective. *Z*-Steps were acquired every 2 μm with 120 to 200 μm of each slice imaged. Images were acquired in galvo mode with pixel sizes of 300 nm and 2× frame averaging.

### Histopathology

Mice were euthanized with CO_2_ and then perfused with 20 ml of 1× PBS, followed by 20 ml of 4% PFA/PBS. Livers and lungs were harvested and postfixed overnight in 4% PFA/PBS on a shaker. Formalin-fixed samples were embedded in paraffin, cut in 5-μm sections, and stained with hematoxylin and eosin, Picrosirius red, and trichrome by Peninsula Histopathology Laboratory. The Picrosirius red–positive area was morphometrically quantified with FIJI image analysis software (ImageJ). Images were converted to grayscale, and the fibrotic area (Sirius red) was calculated using the total liver section area and Sirius red–positive area calculated by the thresholding method. Hematoxylin and eosin, Sirius red, and trichrome stains were reviewed and evaluated by a blinded UCSF liver pathologist (A.N.M.) for steatosis and lobular inflammation using a histological scoring system for nonalcoholic fatty liver disease. 

### Hydroxyproline assay

For fibrosis quantification, a hydroxyproline assay was performed as described previously (*74*). Briefly, 80 to 200 mg of liver tissue was hydrolyzed in 1.5 ml of 6 mol/L HCl at 110°C overnight. Ten microliters of standards or the hydrolyzed samples were pipetted as triplets into a 96-well optically clear plate with 30 μl of citric acid buffer. One hundred microliters of Chloramine T solution was added and allowed to oxidize for 20 min at room temperature. One hundred microliters of Ehrlich’s Reagent was mixed with the oxidized samples or standards and incubated at 65°C for 20 min. Absorbance was read at 550 nm in a spectrophotometer, and samples were quantified by comparison to the standard curve.

### Serum analysis

Blood was harvested after cardiac puncture and processed for collection of serum, snap frozen, and stored at −80°C until further testing. Bilirubin was measured using the Bilirubin Assay Kit following the manufacturer’s instructions (Sigma-Aldrich, cat. no. MAK126-1KT). ALT was assayed on Roche Diagnostic’s Cobas Mira.

### Total tissue RNA extraction and qPCR

RNA from mouse liver tissue was obtained by homogenizing in TRIzol (Thermo Fisher Scientific, cat. no. 15596018) and extracting RNA using the E.Z.N.A. Total RNA Kit (Omega Bio-Tek, cat. no. R6834-01) following the manufacturer’s instructions. RNA was reverse transcribed using the SuperScript III cDNA synthesis kit (Thermo Fisher Scientific), and cDNA was used as a template for quantitative polymerase chain reaction (qPCR) using Power SYBR Green PCR master mix (Thermo Fisher Scientific). Transcripts were normalized to *Gapdh* expression and relative expression shown as 2−ΔΔCt compared to the average ΔCt of experiment-matched controls. The following primers were used: *Desmin*: 5′-GTTTCAGACTTGACTCAGGCA-3′, 5′-TCTCGCAGGTGTAGGACTGG-3′; Acta2: 5′-CCCAAAGCTAACCGGGAGAAG-3′, 5′-GACAGCACCGCCTGGATA-3′; *Snail*: 5′-CACACGCTGCCTTGTGTCT-3′, 5′-GGTCAGCAAAAGCACGGT T-3′; *Col1a1*: 5′-CCAAGAAGACATCCCTGAAGTCA-3′, 5′-TGCACGTCATCGCACACA-3′; *Epcam*: 5′-GCGGCTCAGAGAGACTCT-3′, 5′-CCAAGCATTTAGACGCCAGTTT-3′; *Tgfb1*: 5′-TGGAGCAACATGTGGAACTC-3′, 5′-GTCAGCAGCCGGTTACCA-3′.

### Mesenchymal-ILC2-γδ T cell cocultures

Lungs from wild-type C57Bl/6 mice were harvested, manually dissociated with a razorblade, and subsequently digested with dispase II (7.5 U/ml), collagenase I (112.5 U/ml), and DNase I (40 μg/ml) in PBS for 30 min at 37°C with gentle agitation. Samples were subsequently passed through 70-μm filters, washed, subjected to red blood cell lysis (PharmLyse; BD Biosciences), and enriched for stromal cells through magnetic bead separation of CD45^+^ and CD31^+^ cells before final suspension in FACS buffer [PBS, 3% FBS, penicillin (50 U/ml), and streptomycin (50 μg/ml)]. Sorted AFs (CD45^−^, EpCAM^−^, PDGFRα^+^ Sca1^+^) were seeded in flat-bottom 96-well plates in 200 μl of DMEM [supplemented with 10% FBS, 1× GlutaMAX, penicillin (50 U/ml), and streptomycin (50 μg/ml)] at a density of 6000 to 10,000 cells per well and allowed to form monolayers over 7 to 8 days. At culture day 3, TGF-β1 was added to relevant wells at a concentration of 10 ng/ml to allow for MF differentiation. Lungs from IL-33–injected, Il5tdtomato^Cre/+;^ Rosa26^RFP/+^ mice were harvested for ILC2 purification, and lungs from wild-type C57Bl/6 or RORγt-GFP mice were harvested for γδ T cell purification, subsequently manually dissociated (Miltenyi GentleMacs), and digested with Liberase (0.2 wU/ml) and DNase I (40 μg/ml) in PBS for 30 min at 37°C with gentle agitation. Samples were manually dissociated (Miltenyi GentleMacs) a second time following digestion, passed through 70-μm filters, washed, and subjected to red blood cell lysis (PharmLyse; BD Biosciences) before final suspension in FACS buffer [PBS, 3% FBS, penicillin (50 U/ml), and streptomycin (50 μg/ml)]. Wells containing stromal monolayers were washed with PBS, and then sorted ILC2s (lin^−^, CD45^+^, CD3^−^, CD4^−^, RFP^+^) and/or γδ T cells (lin^−^, CD45^+^, CD3^+^, TCRβ^−^, TCRγδ^+^, RORγt-GFP^+^) were seeded onto the stromal monolayers or cultured with only DMEM at a total volume of 200 μl at a density of 6000 to 8000 ILC2s and/or γδ T cells per well. After 6 to 7 days of culture, supernatants were collected, and cells were liberated; stained for viability dye, CD45, CD3, TCRγδ, Sca-1, and PDGFRα; analyzed; and enumerated using CountBright Absolute counting beads (Life Technologies) by flow cytometry.

### Image analysis and quantification

The Imaris Bitplane 9.7.2 software package (Andor Technology PLC, Belfast, Northern Ireland) was used for all 3D image analysis, and *Z*-stack images were rendered in 3D and quantitatively analyzed using the Bitplane Imaris version 9.5 software package (Andor Technology PLC, Belfast, Northern Ireland). To parse PDGFRα subsets, a colocalization channel was made between PDGFRα and anti–IL-33. PDGFRαGFP^+^ stromal cells and IL-5^+^, IL-17A, and RORγt^+^ lymphocytes were annotated using the Imaris spot’s function on the basis of the fluorescent reporter signal and using the Ortho slicer function to visualize size, morphology, and nuclear staining (DAPI). To localize IL-5^+^, IL-17A^+^, and RORγt^+^ lymphocytes with different stromal cell types and bile ducts, 3D reconstructions of structures labeled for Col1 and CD326 (EpCAM) were generated, followed by calculation of 3D distances between lymphocytes and Col1^+^ surfaces using the Imaris Distance Transform Matlab XTension, and volumetric decile calculations were performed using a Matlab-based Imaris XTension. Col1-associated lymphocytes were defined as spots localized <60 μm from stained Col1 and <25 μm from the Col1a2creERT2; Rosa26^Tdt-Ai14^ surface and parenchymal lymphocytes as >60 μm from stained Col1 surfaces. Periportal lymphocytes were defined as spots localized <60 μm from the SMA^+^ smooth muscle surface, collagen I^dim^ surface, and glutamine synthetase^+^ surfaces.

### Slide preparation for spatial transcriptomics

Five-micrometer sections were cut from the formalin-fixed, paraffin-embedded blocks of two liver tissue samples from each of the four mouse groups. The sections were mounted on Leica BOND Plus slides, ensuring that the tissue sections were properly positioned within the scan area of the GeoMx DSP instrument. The slides were baked at 60°C for 30 min, followed by deparaffinization and rehydration. RNA targets were retrieved and exposed by incubating the slides in 1× tris-EDTA (pH 9) for 20 min, followed by 15-min incubation at 37°C in proteinase K (1 μg/ml; Invitrogen, no. AM2548). The Whole Transcriptome Atlas probe was applied to each slide and hybridized overnight at 37°C in a RapidFISH Slide Hybridizer. The next day, the slides were stained for 1 hour at room temperature with optimally diluted solutions of morphology markers including SYTO83 (Invitrogen, no. S11364), CD45 (Cell Signaling, no. 35154), COL1A1 (Cell Signaling, no. 72827S), and α-SMA (Abcam, no. ab184875).

### Region of interest selection

The stained slides were scanned using the GeoMx DSP instrument with four detection parameters: SYTO83 with Cy3/568 nm, CD45 with Texas Red/615 nm, COL1A1 with Cy5/666 nm, and α-SMA with fluorescein isothiocyanate/525 nm. Regions of interest (ROIs) were selected on the basis of these markers to select the specific area including fibrotic tracts, parenchymal regions, and adventitial regions. A total of 66 ROIs were selected, an average of 1616 nuclei per ROI were captured via ultraviolet illumination, and cleaved oligos were collected to the DSP collection plate.

### Library preparation and sequencing

PCR amplification and indexing were performed on the samples using 2 μl of PCR master mix, 4 μl of primers from the GeoMx Seq Code Pack Plate B, and 4 μl of resuspended DSP aspirate. PCR products were pooled, and AMPure XP beads were added at a 1.2× ratio to the final pooled volume for purification. The library quality was checked with the Agilent 4200 TapeStation system, with an amplicon peak of 169 bp (base pairs). Sequencing was performed at a depth of 100 reads/μm^2^ on an Illumina Novaseq 6000 using paired-end reads with 2 × 151 bp. Fastq files were converted to Digital Count Conversion (DCC) files using the GeoMx NGS Pipeline (version 2.0.0.16).

### Spatial transcriptomics analysis

The converted GeoMx data (DCC files) were imported into R studio (version 1.1.456, R version 4.3.2) for bioinformatics analysis primarily using the R packages GeoMxTools (version 3.5.0) and NanoStringNCTools (version 1.10.0) (*99*). The following filters were applied: at least 1000 mRNA reads per ROI, ROIs with at least 80% of reads (trimmed, stitched, and aligned), minimum sequencing saturation of 50%, negative control count threshold of 1 to 9000, minimum nuclei count of 100, and minimum ROI area of 5000 μm^2^. All 66 ROIs passed these filters. Next, we removed probes on the basis of the calculation on negative control probes, where a probe is considered an outlier according to Grubb’s test of at least 20% of the ROIs. Subsequently, we calculated the limit of quantification for each ROI on the basis of the distribution of negative control probes and filtered for ROIs with less than 10% of genes detected above the ROI’s limit of quantification. This results in a final amount of 12,566 features across 66 ROIs. We then normalized the data with the “normalize” function using the third quantile approach and calculated a tSNE on the log_2_-transformed normalized data. Because four data points did not group together with the other samples from the same genotype, we manually removed them from the dataset. Last, differential expression analysis was performed using a linear mixed-effect model across different conditions (IL-5Cre control versus IL-5Cre CCl_4_, IL-5Cre CCl_4_ versus IL-5–deleter CCl_4_, and IL-5–deleter CCl_4_ versus double-deleter CCl_4_). Models were adjusted for sex and test slide as a random intercept. Differentially expressed genes with an FDR (false discovery rate) of less than 0.0001 were additionally visualized in a heatmap using the pheatmap package by plotting the log_2_ of the quantile-normalized data. Rows and columns were clustered using the UPGMA (unweighted pair group method with arithmetic mean) method, with distances computed as 1 − Pearson correlation. Volcano plots were also generated to visualize genes with significant differential expression using the EnhancedVolcano (version 1.20.0) R package (*100*). Subsequently, we carried out deconvolution analysis using the SpatialDecon R package (*101*). The cell profile matrix of the ImmuneAtlas study (*102*) was used using the “download_profile_matrix” function. We used the processed data from previous NanoString analysis as input and the “spatialdecon” function to run deconvolution analysis on the ROIs across each condition. For multigene score analysis and visualization, DCC files were converted into a Seurat object and processed using Seurat (version 5.2.1). Multigene scores were generated as follows using Seurat’s AddModuleScore function. AF scores were calculated using all differentially expressed markers [*q* < 0.05, log_2_ FC (fold change) > 0] of AFs (*Il33^+^, Pi16^+^*) in an in-house single-cell RNA sequencing dataset derived from healthy and influenza-infected lungs. MF scores were calculated using all differentially expressed markers (*q* < 0.05, log_2_ FC > 0) of *Lrrc15*^+^ MFs from Buechler *et al.* (*65*). Pan-fibroblast scores were calculated using the generic fibroblast genes *Col1a1* and *Col1a2*, *Sparc*, *Col3a1*, *Dcn*, *Dpt*, and *Pdgfra* (*65*). Pericentral scores were calculated using *Igfbp1*, *Nt5e*, *Cyp3a4*, *Adh4*, *Glul*, and *Bche*; periportal scores were calculated using *Igf1*, *Cdh1*, *Cyp7a1*, *Hsd3b7*, *Hal*, *Cps1*, and *Hmgcs1* (*66*). SAM scores were generated using *Trem2*, *Cd9*, *Spp1*, *Gpnmb*, *Fabp5*, and *Cd63* (*58*). Violin plots were generated to visualize all module scores after splitting by genotype, condition, and/or microanatomical region.

### Statistical analysis

All data are expressed as the means ± standard error of the mean (SEM), unless otherwise noted. Comparisons between two groups were analyzed by using unpaired two-tailed Student’s *t* tests, and multiple comparisons were analyzed by a one-way analysis of variance (ANOVA) with Tukey’s multiple comparisons test for normally distributed data, unless otherwise noted (Prism, GraphPad Software, La Jolla, CA), with *P < 0.05, **P < 0.01, ***P < 0.001, and ****P < 0.0001. When possible, results from independent experiments were pooled. Data points reflect individual biological mouse replicates for flow analysis or individual tissue slices analyzed for confocal imaging.

## Supplementary Materials

### The PDF file includes:

Figs. S1 to S7 Legends for movies S1 to S5 References
