# Supplementary material for: Type 2 lymphocytes restrict type 3 lymphocytes during liver fibrosis and colocalize in fibroblast niches
Source: Sci Adv. 2026 Mar 11;12(11):eaea6805. doi: 10.1126/sciadv.aea6805 (PMC12978224; doi:10.1126/sciadv.aea6805)
Supplement: Supplementary file 1 — Figs. S1 to S7 Legends for movies S1 to S5 References [file sciadv.aea6805_sm.pdf]

Science Advances
AAAS

Supplementary Materials for

Type 2 lymphocytes restrict type 3 lymphocytes during liver fibrosis and colocalize in fibroblast niches

Julia Sbierski-Kind *et al.*

Corresponding author: Ari B. Molofsky, ari.molofsky@ucsf.edu

Sci. Adv. **12**, eaea6805 (2026)
DOI: 10.1126/sciadv.aea6805

The PDF file includes:

Figs. S1 to S7
Legends for movies S1 to S5
References

Other Supplementary Material for this manuscript includes the following:

Movies S1 to S5

Supplemental information

Supplementary Figure 1

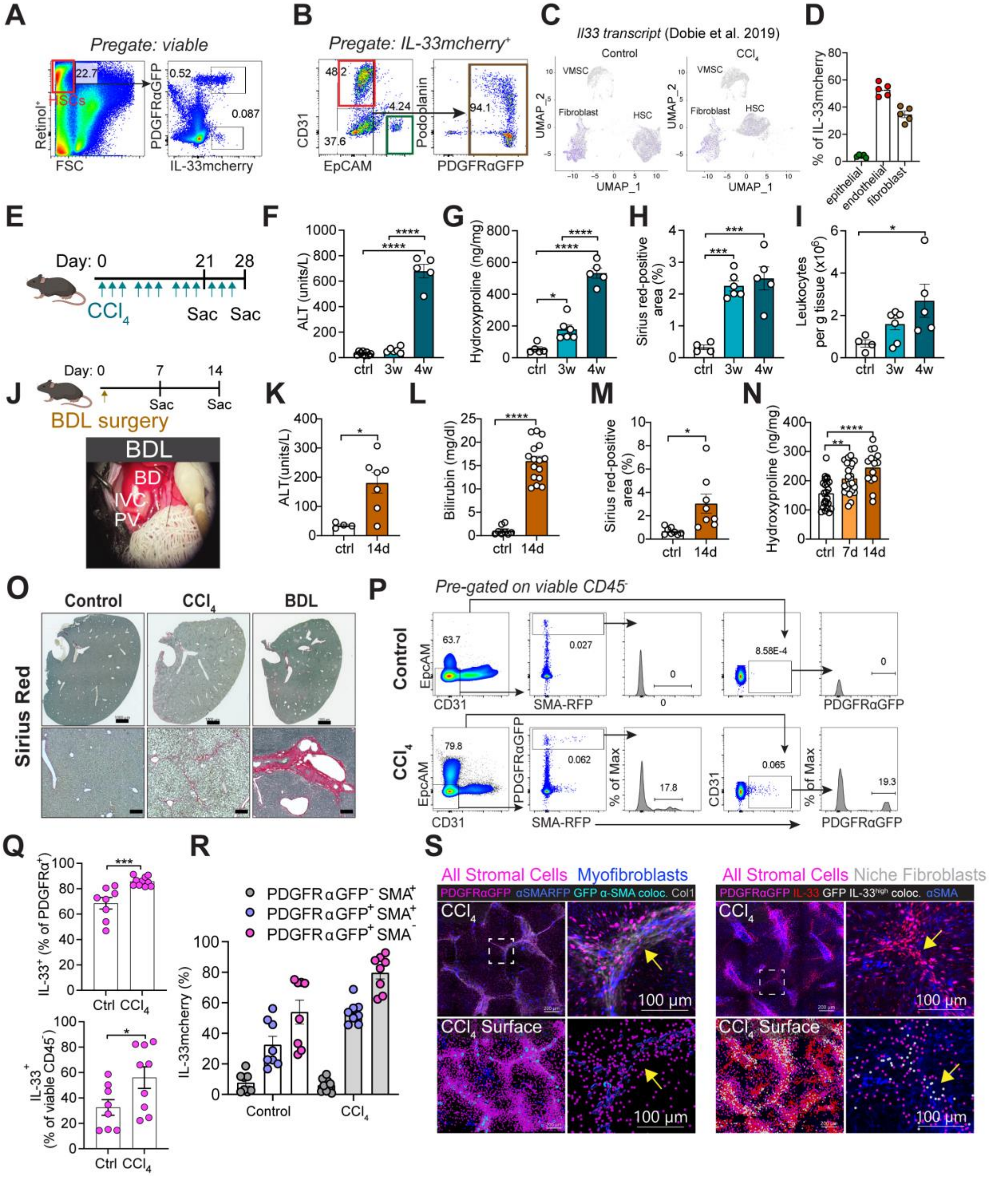

Figure S1. **Models of liver fibrosis and impact on stromal cell topography (related to Figure 1). (A)** Representative flow cytometry plots from livers of 4-week CCl_4-treated PDGFRαGFP; IL-$33^{mcherry/+}$ reporter mice, showing expression of PDGFRαGFP and IL-33mcherry in hepatic stellate cells (HSCs). HSCs are selected based on the UV light excitation of retinol (vitamin A) (*103*). Data are representative of three repeats, N ≥ 5 mice/ group. (**B, D)** Representative flow cytometry plots (B) and quantification (D) from livers of 4-week CCl_4-treated PDGFRαGFP; IL-$33^{mcherry/+}$ reporter mice, showing percent epithelial ($EpCAM^+$), endothelial cells ($CD31^+$), and fibroblasts ($CD31^-EpCAM^-PDGFR\alpha GFP^+$) of $IL33mcherry^+$ non-hematopoietic $CD45^-$ cells. Data are representative of three repeats, N ≥ 5 mice/ group. (**C)** Expression of *Il33* in single cell RNA sequencing of mesenchymal cells from control and fibrotic (6 weeks CCl_4) mouse livers; clustered into three subpopulations. Fibroblasts; HSC, hepatic stellate cells; VSMC, vascular smooth muscle cells (generated from (Dobie et al.; 2019) (*41*) dataset). (**E)** Schematic showing CCl_4 administration schedule in IL-$33^{mcherry/+}$ reporter mice injected intraperitoneally (i.p.) with 0.5µl CCl_4/g BW three times per week for 3 or 4 weeks (3w, 4w), relevant to F-I. (**F-I)** Quantification of ALT (F), hepatic Hydroxyproline levels (G), Sirius red positive staining (H), total leukocytes (I) in control mice and CCl_4-treated mice at the indicated timepoints. Total N ≥ 4 mice/ group. (**J)** Schematic of bile duct ligation (BDL) surgery in PDGFRαGFP; $IL33^{mcherry/+}$ reporter mice for time course analysis, relevant to K-N. Bile duct (BDL), Portal vein (PV), Inferior vena cava (IVC). (**K-N)** Quantification of ALT (K), Bilirubin (L), Sirius red positive staining (M), and hepatic Hydroxyproline levels (N) in control mice and bile duct ligated mice at the indicated timepoints. Pooled data from 3 independent experiments, N ≥ 4 mice/ group. (**O)** Representative Sirius Red staining of 5µm paraffin liver sections from IL-33 $^{mcherry/+}$ reporter mice at steady state, at day 28 of CCl_4 treatment, and at day 14 post BDL surgery. Higher magnification images show detailed

morphology of collagen deposition. Scale Bars: 100µm (10X). Images are representative of 3 repeat experiments with N = 3-10 mice per group. (**P)** Representative flow cytometry plots from liver of PDGFRαGFPSMARFP reporter mice treated with vehicle or CCl_4 for 4 weeks and representative histograms of αSMA expression on PDGFRαGFP$^+$ fibroblasts. Data are representative of 2 independent experiments, N ≥ 4 mice/group. (**Q)** Flow cytometry quantification, showing liver IL-33$^+$ cells as percent of PDGFRαGFP$^+$ fibroblasts (above) and as percent of viable CD45$^-$ cells (below) from PDGFRαGFP; IL-33$^{mcherry/+}$ mice treated with vehicle or CCl_4 for 4 weeks. Pooled data from 2 independent experiments, N ≥ 4 mice/ group. (**R)** Quantification of percentages IL-33mcherry expression of different fibroblast subsets defined by PDGFRαGFP and αSMA. Bar graphs are representative of 4 repeat experiments with N ≥ 5 mice/ group. (**S)** Representative thick section confocal images of livers from 4-week CCl_4-treated PDGFRα-H2B-eGFP; FoxP3DTR/αSMA-RFP mice (left) and PDGFRαGFP; IL-33$^{mcherry/+}$ reporter mice (right). Antibody stains are indicated, and PDGFRαGFP-IL-33mcherry co-localization is highlighted in white. Images are representative of three or more mice per group. Scale bars represent 200µm unless otherwise indicated.

Bar graphs indicate mean (±SE). Student`s t-test (K-M, Q) or one-Way ANOVA with Tukey post-test (F-I, N). *p ≤ 0.05, **p ≤ 0.01, ***p ≤ 0.001, ****p ≤ 0.0001.

Supplementary Figure 2

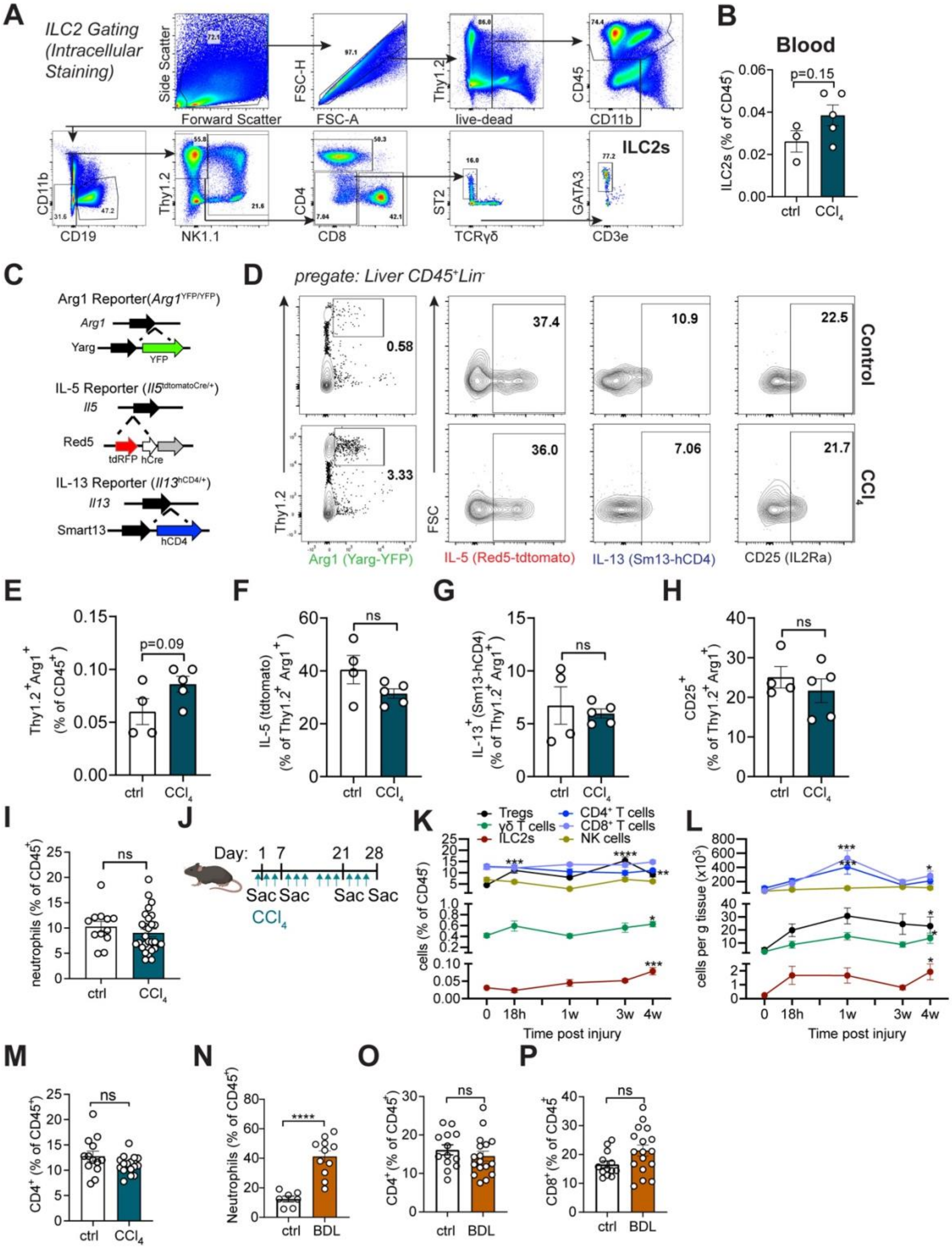

Figure S2. **Type 2 and type 3 lymphocytes expand in liver damage and fibrosis (related to Figure 2). (A)** Representative flow gating scheme for liver ILC2s (viable $CD45^{+}Lin^{-}GATA3^{+}ST2^{+}$) and T cells. (**B)** Blood ILC2s (viable $CD45^{+}Lin^{-}GATA3^{+}ST2^{+}$) in control mice and 4-week CCl_4-treated mice. Total N ≥ 3 mice/ group. (**C)** Schematic of the IL-5^{+} lymphocyte lineage tracker mice (IL-5tdtomato-Cre; Rosa26RFP), *Il13*^{Smart} (Smart13; B6.129S4[C]-*Il13*$^{tm2.1Lky}$/J; 031367), and *Arg1*$^{RFP\text{-}CreERT2}$ mice. (**D-H)** Representative flow cytometry plots (D) and quantification of ILC2 ($Lin^{-}Thy1.2^{+}Arg^{+}$) (E) expression of IL-5RFP (F), IL-13 (G), and CD25 (H) in livers of control mice and 4-week CCl_4-treated mice on Arg1 (Yarg); R5 (IL-5); S13 (IL-13) combined triple-reporter (YRS) background. Total N ≥ 4 mice/ group. (**I)** Flow cytometry quantification showing percentages of neutrophils in livers from IL-$33^{mcherry/+}$ reporter mice treated with vehicle or CCl_4 for 4 weeks. Pooled data from 4 independent experiments, N ≥ 5 mice/ group. (**J)** Schematic showing CCl_4 administration schedule in IL-$33^{mcherry/+}$ reporter mice injected intraperitoneally (i.p.) with 1μl CCl_4/g body weight (BW) once (18h) or with 0.5μl CCl_4/g BW three times per week for 1, 3, or 4 weeks (1w, 3w, 4w). (**K and L)** Time course showing percentage (K) and total numbers (L) of $CD8^{+}$ T cells, $CD4^{+}$ T cells, NK cells, ILC2s, Tregs, and χδ T cells, in livers from IL-$33^{mcherry/+}$ reporter mice treated with vehicle or CCl_4 for the indicated duration. Pooled data from 3 independent experiments, N ≥ 6 mice/ group. **(M)** Flow cytometry quantification, showing percent $CD4^{+}$ T cells from IL-33mchery reporter mice treated with vehicle or CCl_4 for 4 weeks. Pooled data from 3 independent experiments, N ≥ 4 mice/ group. **(N-P)** Flow cytometry quantification, showing percent neutrophils (N), $CD4^{+}$ T cells (O), and $CD8^{+}$ T cells (P) in livers from IL-5 Cre mice 14 days post-BDL surgery or sham surgery. Pooled data from 3 independent experiments, N ≥ 2 mice/ group.

Bar graphs indicate mean (±SE). Student`s t-test (B, E-I, M-P); one-way ANOVA with Tukey`s post-test per timepoint (K, L). ns= not significant, ****$p \leq 0.0001$.

Supplementary Figure 3

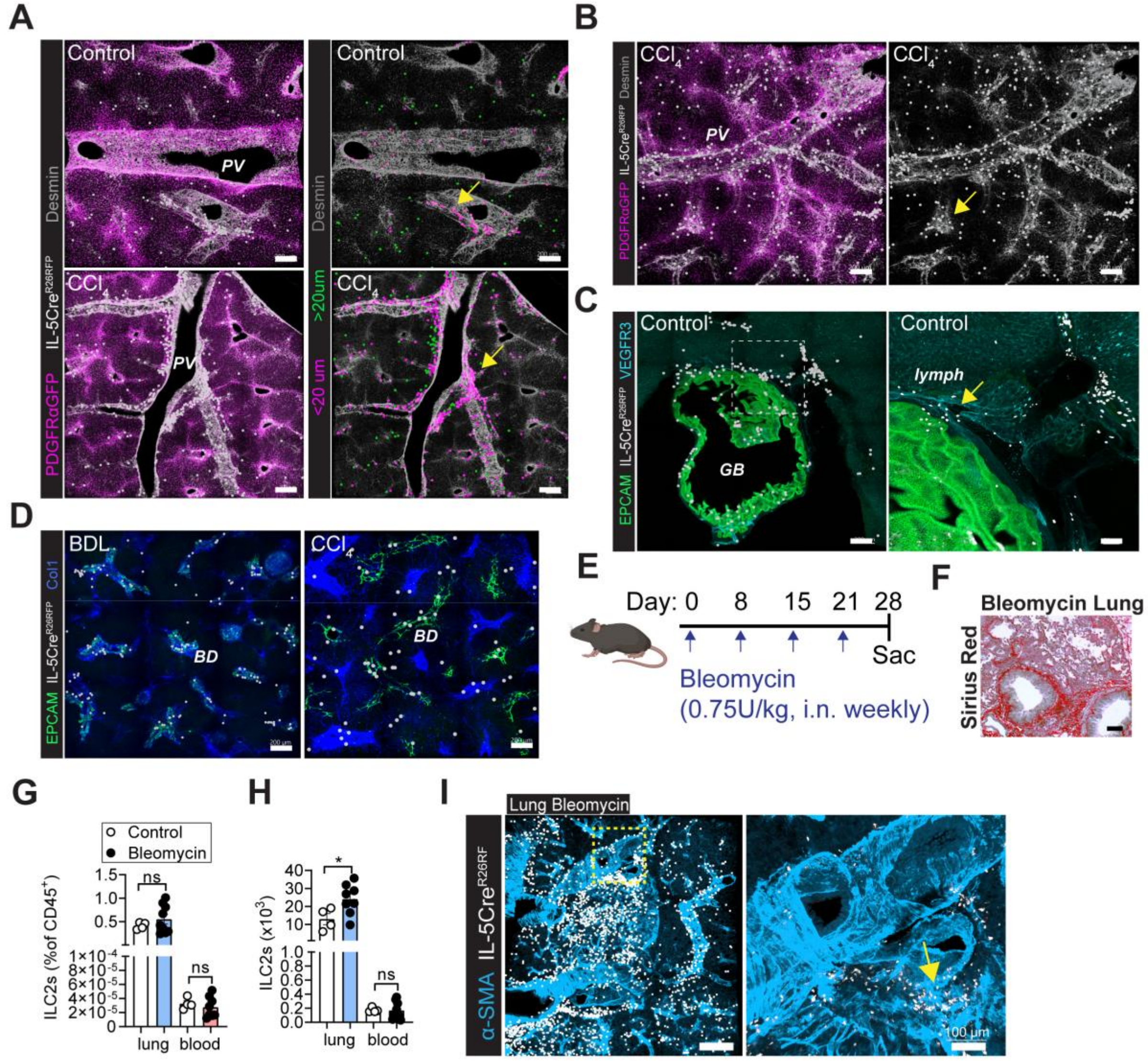

Figure S3. **Type 2 lymphocytes localize to periportal regions at steady state and expand into fibrotic tracts with liver fibrosis (related to Figure 3). (A)** Representative confocal imaging of liver from PDGFRαGFP; Il5tdtomato-$^{Cre/+;}$ Rosa26$^{RFP/+}$ mice treated with vehicle or CCl_4 for 4 weeks with surface rendering of IL-5$^+$ lymphocytes and indicated staining. Images are representative of three or more mice. Yellow arrows indicate IL-5$^+$ lymphocytes closer than 20µm to Desmin. Portal vein (PV; zone 1). Scale bars represent 200µm**. (B)** Representative confocal

imaging of liver from PDGFRαGFP; Il5tdtomato-$^{Cre/+;}$ Rosa26$^{RFP/+}$ mice treated with CCl_4 for 4 weeks with surface rendering of IL-5$^+$ lymphocytes and indicated staining. Images are representative of three or more mice. Yellow arrows indicate IL-5$^+$ lymphocytes. Scale bars represent 200µm**. (C)** Representative confocal imaging of gallbladder (GB) and liver from naïve IL-5$^+$ lymphocyte lineage tracker mice with staining for EpCAM (epithelial duct cells) and VEGFR3 (lymphatics). Higher magnification highlighting accumulation of IL-5$^+$ lymphocytes around the bile ducts. Image is representative of three mice. Yellow arrows indicate IL-5$^+$ lymphocytes. Scale bars represent 200µm (left) and 50µm (right). **(D)** Representative confocal thick section imaging of liver from IL-5$^+$ lineage tracker mice 14 days post BDL surgery or treated with CCl_4 for 4 weeks, and surface rendering of IL-5$^+$ lymphocytes and indicated staining. Images are representative of three or more mice. Bile duct (BD). Scale bars represent 200µm**. (E)** Schematic showing Bleomycin administration schedule in IL-5 reporter mice subjected to weekly intranasal applications of Bleomycin (0.75U/kg), relevant to F-I. (**F)** Representative Sirius red staining from IL-5tdtomato-Cre mice treated with Bleomycin for 4 weeks. Images are representative of $N \geq 4$ mice/ group. **(G and H)** Flow cytometry quantitation of percent (G) and total numbers (H) of ILC2s in lung and blood from PDGFRαGFP; IL-33$^{mcherry/+}$ mice treated weekly with Bleomycin or PBS (intranasal application) for 4 weeks. Total $N \geq 4$ mice/ group. (**I)** Representative confocal imaging of cleared thick lung sections with surface analysis for IL-5RFP$^+$ lymphocytes from Il5-tdtomato-Cre mice treated weekly with Bleomycin (intranasal application) for 4 weeks. Images are representative of $N \geq 4$ mice/ group. Yellow arrow indicates IL-5$^+$ lymphocytes. Scale bars represent 200µm (left) and 100µm (right).

Bar graphs indicate mean (±SE). Student`s t-test for G and H, ns= not significant, $*p \leq 0.05$.

Supplementary Figure 4

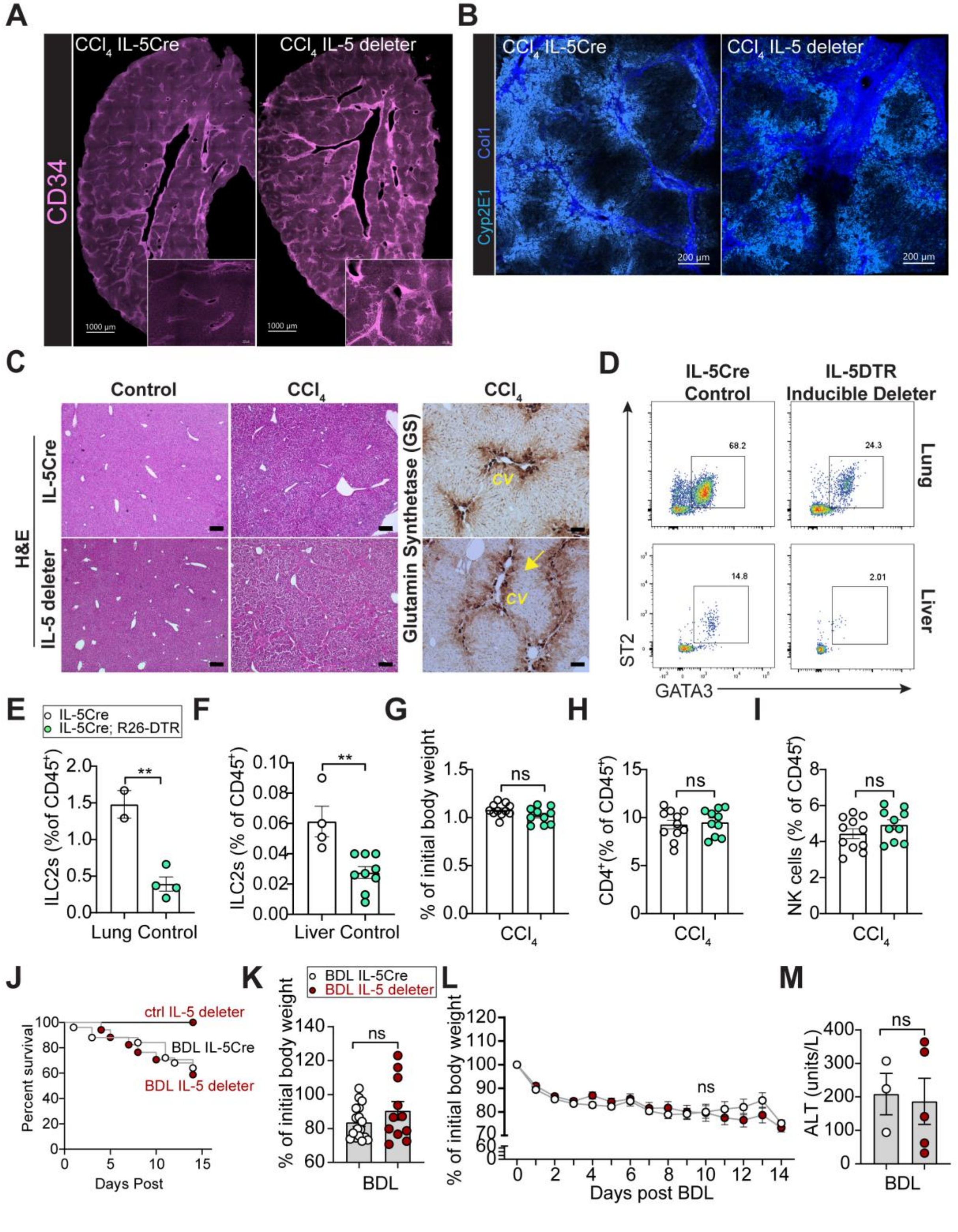

Figure S4. **Loss of IL-5$^+$ lymphocytes worsens hepatic fibrosis (related to Figure 5). (A)** Representative confocal liver tissue sections from control or 4-week CCl_4-treated Il5-tdtomato-Cre mice and IL-5 deleter (*Il5*$^{Cre\text{-}RFP/Cre\text{-}RFP}$;*R26R*$^{DTA/DTA}$) mice with staining for CD34. Images shown are representative of 3-4 mice/group. Scale bars represent 1000µm**. (B)** Representative confocal thick section imaging of liver from IL-5tdtomato-Cre and IL-5 deleter mice with staining for Collagen I and Cyp2E1 (marker of pericentral hepatocytes). Images shown are representative of 3-4 mice/group. Scale bars represent 200µm**. (C)** Representative hematoxylin-eosin (H&E) stain and glutamin synthetase (GS) immunohistochemistry from IL-5tdtomato-Cre and IL-5 deleter mice treated with vehicle or CCl_4 for 4 weeks. Central vein (CV; zone 3). Images are representative of 3 independent experiments, N ≥ 8 mice/ group. Scale bars represent 200µm (left) or 100µm (right)**. (D-F)** Representative flow cytometry plots of ILC2s (Lin$^-$ Thy1.2$^+$GATA3$^+$ST2$^+$) (D) and flow quantitation of ILC2 percentages in lung (E) or liver (F) from either IL-5tdtomato-Cre or IL-5^{DTR} (*Il5*$^{Cre\text{-}RFP/Cre\text{-}RFP}$;*R26R*$^{DTR/DTR}$) treated with CCl_4 for 4 weeks. Pooled from 2 independent experiments, N = 4-10 mice/ group. (**G-I)** Percent of initial body weight before CCl_4 treatment (G), percent CD4$^+$ T cells (H), and percent NK cells (I) in livers of 4-week CCl_4-treated IL-5tdtomato-Cre and IL-5^{DTR} mice. Pooled data from 2 independent experiments, N ≥ 5 mice/ group. (**J-M)** Kaplan-Meier-survival curves (J), body weight loss in percentage of initial body weight (K and L) after bile duct ligation (BDL), and ALT levels (M) from bile duct ligated Il5-tdtomato-Cre mice and IL-5 deleter mice. Data for survival curves are pooled from 3 independent experiments, N = 25 BDL Il5-tdtomato-Cre mice, N = 17 BDL IL-5 deleter mice, N = 10 control Il5-tdtomato-Cre mice, and N = 4 control IL-5 deleter mice. Other data are pooled from 3 independent experiments with N = 3-13 mice/group. Bar graphs indicate mean (±SE). Kaplan-Meier survival curves are compared using the log-rank (Mantel-Cox) analysis

(J). Student`s t-test (E-I, K, M) or two-way ANOVA with Sidak`s post-test per timepoint (L). ns = not significant, $^{**}p \leq 0.01$.

Supplementary Figure 5

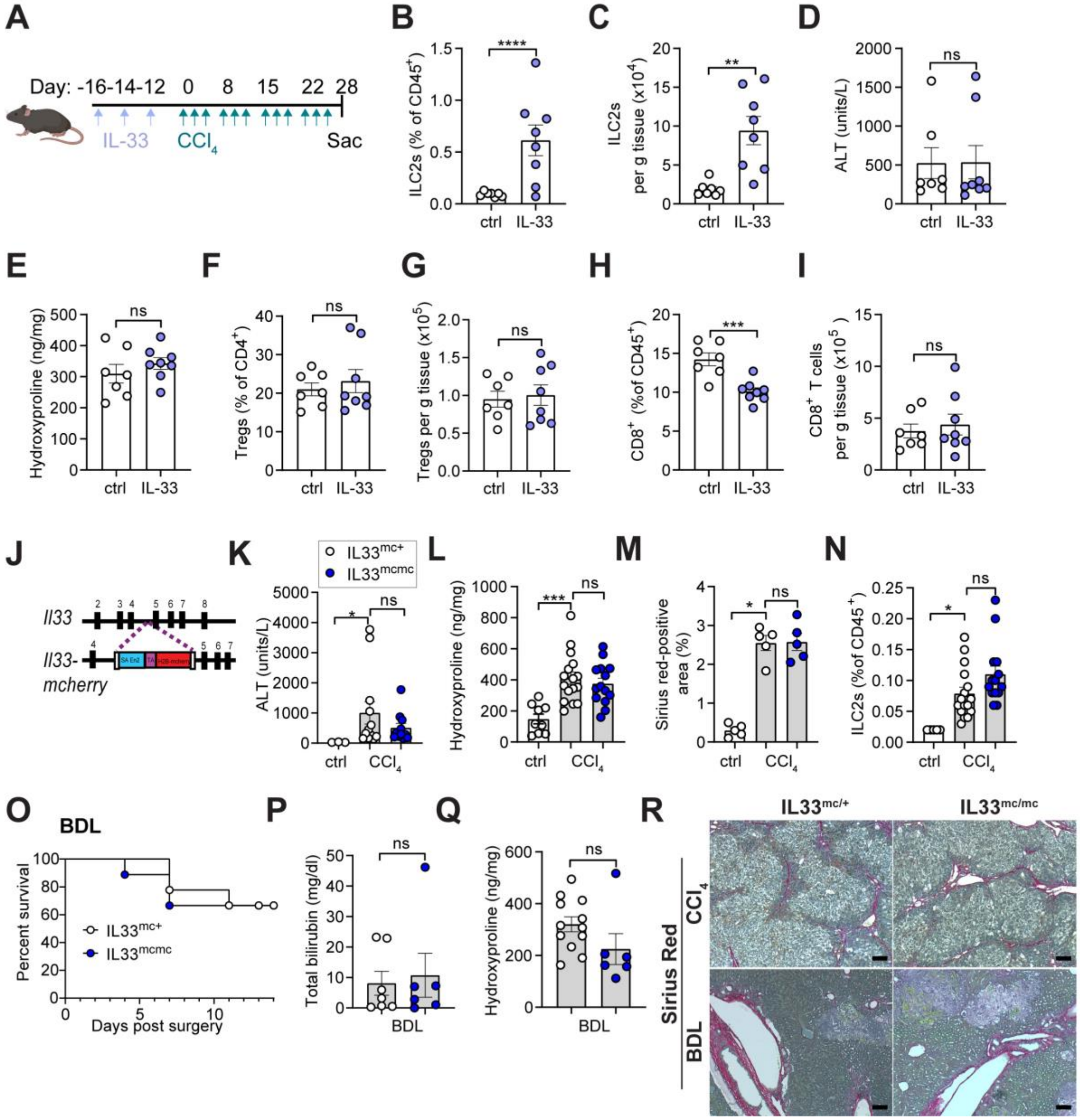

Figure S5. **Neither ILC2 expansion nor IL-33 signalling impact degree of hepatic fibrosis. (A)** Schematic of CCl_4 and IL-33 treatment (500ng i.p.) for IL-5^+ lineage tracker mice (IL-5tdtomato-Cre; Rosa26RFP). (**B and C)** Flow cytometry quantitation of percent (B) and total numbers (C) of ILC2s in control and IL-33-treated IL-5^+ lineage tracker mice after 4 weeks of CCl_4 treatment. Total N ≥ 7 mice/ group. (**D and E)** Quantification of ALT (D) and hepatic Hydroxyproline levels (E) in control and IL-33-treated IL-5^+ lineage tracker mice after 4 weeks of CCl_4 treatment. Total N ≥ 7 mice/ group. (**F-I)** Flow cytometry quantitation of percent (F) or total numbers (G) of Tregs and percent (H) or total numbers (I) of $CD8^+$ T cells from control and IL-33-treated IL-5^+ lineage tracker mice after 4 weeks of CCl_4 treatment. Total N ≥ 7 mice/ group. (**J)** Diagram of the construction for the IL-33-H2B-mcherry nuclear localization IL-33 reporter. (**K-M)** Quantification of ALT (K), hepatic Hydroxyproline (L), and Sirius red staining (M) from IL-$33^{mcherry/+}$ and IL-$33^{mcherry/mcherry}$ mice treated with CCl_4 for 4 weeks or controls. Pooled data from 3 independent experiments, N ≥ 3 mice/ group. (**N)** Flow cytometry quantification, showing percent of ILC2s in livers from IL-$33^{mcherry/+}$ and IL-$33^{mcherry/mcherry}$ mice treated with CCl_4 for 4 weeks or controls. Pooled data from 3 independent experiments, N ≥ 3 mice/ group. (**O)** Kaplan–Meier survival curves after bile duct ligation (BDL). Data are pooled from 2 independent experiments, N ≥ 6 mice/ group. (**P and Q)** Quantification of total bilirubin (P) and Hydroxyproline (Q) from IL-$33^{mcherry/+}$ and IL-$33^{mcherry/mcherry}$ mice 14 days post BDL surgery. Pooled from 2 independent experiments, N ≥ 6 mice/ group. (**R)** Representative Sirius red staining from IL-$33^{mcherry/+}$ and IL-$33^{mcherry/mcherry}$ mice treated with CCl_4 for 4 weeks or 14 days post BDL surgery, as indicated. Images are representative of ≥ 3 mice/ group. Scale bars represent 100μm**.** Bar graphs indicate mean (±SE). Student`s t-test (B-I, K-N, P, Q). Kaplan-Meier survival curves are compared using

the log-rank (Mantel-Cox) analysis (O). ns= not significant, *$p \leq 0.05$, **$p \leq 0.01$, ***$p \leq 0.001$, ****$p \leq 0.0001$.

Supplementary Figure 6

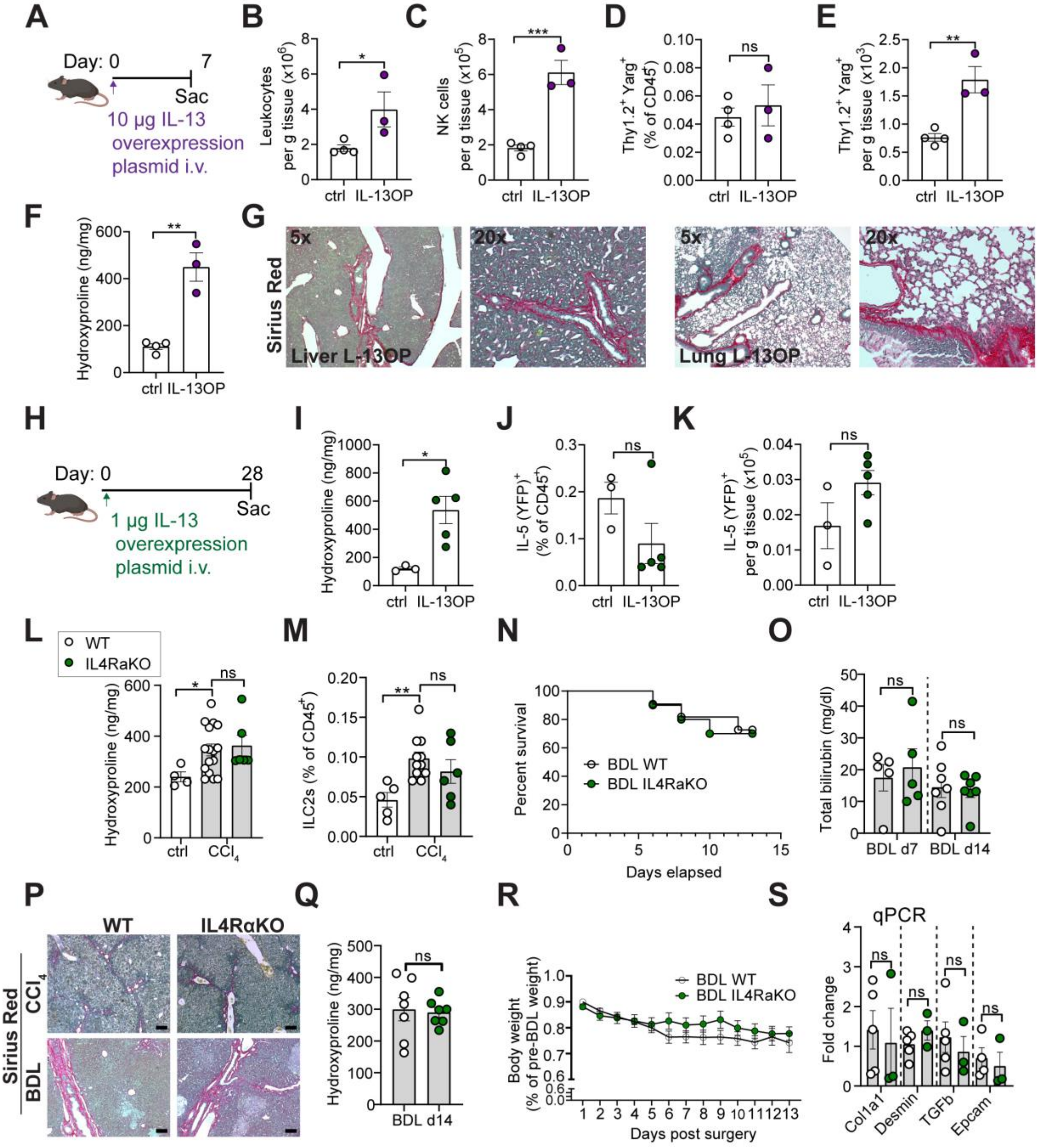

Figure S6. **The IL-4/IL-13 receptor does not regulate hepatic fibrosis in different models of liver injury. (A)** Schematic showing intravenous (i.v.) treatment with 10µg IL-13 overexpressing plasmid or PBS in Arg1 (Yarg); R5 (IL-5); S13 (IL-13) combined triple-reporter (YRS) mice. Mice were harvested after 7 days. **(B-E)** Flow cytometry quantitation of total leukocyte numbers (B), total numbers of NK cells (C), percent (D), and total numbers of ILC2s ($Lin^{-}Thy1.2^{+}Arg1^{+}$) (E) in control and $_{plasmid}$IL-13-treated YRS mice. Total N ≥ 3 mice/ group. **(F and G)** Quantification of Hydroxyproline in control and $_{plasmid}$IL-13-treated YRS mice (F) and representative Sirius red staining from liver and lung of $_{plasmid}$IL-13-treated YRS mice (G). Images are representative of three or more mice per group. **(H)** Schematic showing intravenous (i.v.) treatment with 1µg IL-13 overexpressing plasmid or PBS in mice. Mice were harvested after 4 weeks. (**I)** Quantification of Hydroxyproline levels in control and $_{plasmid}$IL-13-treated mice. Total N ≥ 3 mice/ group. **(J and K)** Flow cytometry quantification of percent (J) and total numbers (K) of $IL\text{-}5^{+}$ lymphocytes in control and $_{plasmid}$IL-13-treated mice. Total N ≥ 3 mice/ group. (**L and M)** Quantification of Hydroxyproline (L) and flow cytometry quantification, showing percent of ILC2s (M) in liver from wild-type (WT) or IL-4/IL-13-deficient mice treated with vehicle or CCl_4 for 4 weeks. Data are pooled from 2 independent experiments, N ≥ 5 mice/ group. (**N)** Kaplan–Meier survival curves after bile duct ligation (BDL) from wild-type and IL-4/IL-13-deficient mice. Data are pooled from 2 independent experiments, N ≥ 7 mice/ group. (**O)** Quantification of bilirubin from WT and IL-4/IL-13-deficient mice 14 days post BDL surgery. Data are pooled from 2 independent experiments, N ≥ 5 mice/ group. **(P)** Representative Sirius red staining from WT and IL-4/IL-13-deficient mice treated with CCl_4 for 4 weeks or 14 days post BDL surgery, as indicated. Images are representative of seven or more mice per group. Scale bars represent 100µm**. (Q)** Quantification of Hydroxyproline from WT and IL-4/IL-13-deficient mice 14 days post BDL

surgery. Data are pooled from 2 independent experiments, N $\geq$ 7 mice/ group. (**R)** Body weight loss of WT and IL-4/IL-13-deficient mice post BDL surgery. Data are pooled from 2 independent experiments, N $\geq$ 7 mice/ group. (**S)** Total liver gene expression of *COL1A1, DES, TGFB1*, and *EPCAM* normalized to *GAPDH* expression in control or bile duct ligated WT or IL-4/IL-13-deficient mice. Data are pooled from 2 independent experiments, N $\geq$ 7 mice/ group.

Bar graphs indicate mean (±SE). Kaplan-Meier survival curves are compared using the log-rank (Mantel-Cox) analysis for N. Student`s t-test (B-F, I-K, O, Q, and S), one-way ANOVA with Tukey`s post-test (L, M), or two-Way ANOVA with Sidak post-test (R). ns = not significant, *$p \leq 0.05$, **$p \leq 0.01$, ***$p \leq 0.001$.

Supplementary Figure 7

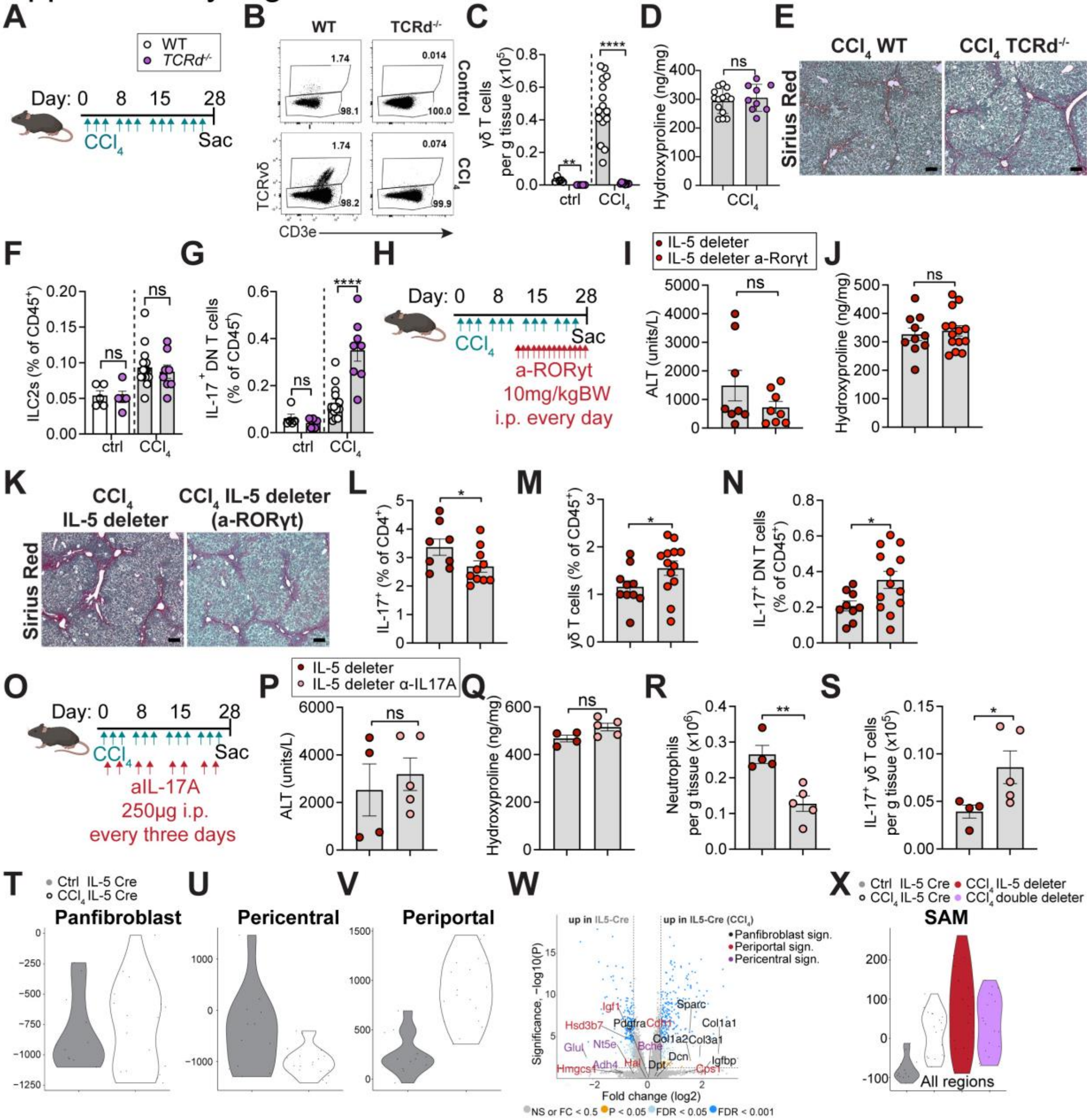

Figure S7. **Redundancy in type 3/17 lymphocytes during hepatic fibrosis (related to Figure 7). (A)** Schematic showing CCl_4 administration schedule in mice injected intraperitoneally (i.p.) with 0.5µl CCl_4/g BW three times per week for 4 weeks (4w), relevant to B-G. (**B and C)** Representative flow cytometry plots of γδ T cells (B) and quantification of total numbers (C) of γδ T cells in wild-type (WT) and *TCRd*$^{-/-}$ mice treated with vehicle or CCl_4 for 4 weeks. Pooled from 2 independent experiments, N ≥ 5 mice/ group. (**D)** Quantification of hepatic Hydroxyproline in WT and *TCRd*$^{-/-}$ mice treated with CCl_4 for 4 weeks. Pooled from 2 independent experiments, N ≥ 5 mice/ group. (**E)** Representative Sirius red staining from WT and *TCRd*$^{-/-}$ mice treated with CCl_4 for 4 weeks. Images are representative of 2 independent experiments with N ≥ 5 mice/ group. Scale bars represent 100µm (**F and G)** Flow cytometry quantification, showing percent of ILC2s (F) and IL-17^{+} DN T cells (G) in livers from WT and *TCRd*$^{-/-}$ mice treated with vehicle or CCl_4 for 4 weeks. Pooled from 2 independent experiments, N ≥ 5 mice/ group. (**H)** Schematic showing treatment strategy for CCl_4 injected intraperitoneally (i.p.) with 0.5µl CCl_4/g BW three times per week for 4 weeks and RORγt antagonist injected with 10mg/kg BW i.p. every day for the last 2 weeks of the experiment with IL-5 deleter (*Il5*$^{\text{Cre-RFP/Cre-RFP}}$*;R26R*$^{\text{DTA/DTA}}$) mice, relevant to data in I-N. (**I and J)** Quantification of ALT levels (I) and hepatic Hydroxyproline (J) in 4-week CCl_4-treated IL-5 deleter mice treated with RORγt antagonist or vehicle. Pooled data from 3 independent experiments, N ≥ 3 mice/ group. (**K)** Representative Sirius red staining from 4-week CCl_4-treated IL-5 deleter mice treated with RORγt antagonist or vehicle. Images are representative of 3 independent experiments with N ≥ 3 mice/ group. Scale bars represent 100µm. (**L-N)** Flow cytometry quantification, showing percent of Th17 cells (L), percent of γδ T cells (M), and percent of IL-7^{+} DN T cells (N) in livers from IL-5 deleter mice treated with RORγt antagonist or vehicle. Pooled data from 3 independent experiments, N ≥ 3 mice/ group. (**O)** Schematic showing CCl_4

and neutralizing anti-IL17A administration in IL-5 deleter mice, injected intraperitoneally (i.p.) with 0.5µl CCl_4/g BW three times per week and with anti-IL-17A antibody every three days throughout the experiment, relevant to P-S. (**P and Q)** Quantification of ALT levels (P) and Hydroxyproline (Q) in livers from IL-5 deleter mice treated with anti-IL-17A antibody or vehicle. Total $N \geq 4$ mice/ group. (**R and S)** Flow cytometry quantification of total numbers of neutrophils (R) and IL-17^+ $\gamma\delta$ T cells (S) in livers from L-5 deleter mice treated with anti-IL-17A antibody or vehicle. Total $N \geq 4$ mice/ group. (**T-V)** Panfibroblast score (7 gene signature (*65*)) (T), Pericentral score (6 gene signature (*66*)) (U), and Periportal score (purple, 7 gene signature (*66*)) (V) in 4-week CCl_4-treated Il5-tdtomato-Cre mice compared to control Il5-tdtomato-Cre mice. (**W)** Volcano plots depict signature genes of Panfibroblast score, Periportal score, and Pericentral score in 4-week CCl_4-treated Il5-tdtomato-Cre mice compared to control Il5-tdtomato-Cre mice across areas. (**X)** Scar-associated macrophages score (6 gene signature) (*58*) in indicated groups. Bar graphs indicate mean (±SE). Student`s t-test (C, D, F, G, I, J, L-N, P-S). ns = not significant, FDR = false discovery rate. $^*p \leq 0.05$, $^{**}p \leq 0.01$, $^{****}p \leq 0.\ 0001$.

Movie S1-Movie S5

Movie S1, Related to Fig 1: Naïve liver IL-33 expressing stroma: serial Z stack sections

Movie S2, Related to Fig 1: CCl_4-treated liver IL-33 expressing stroma: 3D

Movie S3, Related to Fig 3: Naïve gallbladder ILC2s: 3D

Movie S4, Related to Fig 3: CCl_4-treate liver ILC2s in fibrotic tracts: 3D

Movie S5, Related to Fig 3: BDL liver ILC2s in perivascular niches: 3D

REFERENCES

1. R. L. Gieseck, M. S. Wilson, T. A. Wynn, Type 2 immunity in tissue repair and fibrosis. *Nat. Rev. Immunol.* **18**, 62–76 (2018).

2. V. Hernandez-Gea, S. L. Friedman, Pathogenesis of liver fibrosis. *Annu. Rev. Pathol. Mech. Dis.* **6**, 425–456 (2011).

3. X. Fan, A. Y. Rudensky, Hallmarks of tissue-resident lymphocytes. *Cell* **164**, 1198–1211 (2016).

4. F. Annunziato, C. Romagnani, S. Romagnani, The 3 major types of innate and adaptive cell-mediated effector immunity. *J. Allergy Clin. Immunol.* **135**, 626–635 (2015).

5. L. Tortola, A. Jacobs, L. Pohlmeier, F.-J. Obermair, F. Ampenberger, B. Bodenmiller, M. Kopf, High-dimensional T helper cell profiling reveals a broad diversity of stably committed effector states and uncovers interlineage relationships. *Immunity* **53**, 597–613.e6 (2020).

6. M. M. Fort, J. Cheung, D. Yen, J. Li, S. M. Zurawski, S. Lo, S. Menon, T. Clifford, B. Hunte, R. Lesley, T. Muchamuel, S. D. Hurst, G. Zurawski, M. W. Leach, D. M. Gorman, D. M. Rennick, IL-25 induces IL-4, IL-5, and IL-13 and Th2-associated pathologies in vivo. *Immunity* **15**, 985–995 (2001).

7. S. D. Hurst, T. Muchamuel, D. M. Gorman, J. M. Gilbert, T. Clifford, S. Kwan, S. Menon, B. Seymour, C. Jackson, T. T. Kung, J. K. Brieland, S. M. Zurawski, R. W. Chapman, G. Zurawski, R. L. Coffman, New IL-17 family members promote Th1 or Th2 responses in the lung: In vivo function of the novel cytokine IL-25. *J. Immunol.* **169**, 443–453 (2002).

8. K. Moro, T. Yamada, M. Tanabe, T. Takeuchi, T. Ikawa, H. Kawamoto, J. Furusawa, M. Ohtani, H. Fujii, S. Koyasu, Innate production of T_H2 cytokines by adipose tissue-associated c-Kit^+Sca-1^+ lymphoid cells. *Nature* **463**, 540–544 (2010).

9. D. R. Neill, S. H. Wong, A. Bellosi, R. J. Flynn, M. Daly, T. K. A. Langford, C. Bucks, C. M. Kane, P. G. Fallon, R. Pannell, H. E. Jolin, A. N. J. McKenzie, Nuocytes represent a new innate effector leukocyte that mediates type-2 immunity. *Nature* **464**, 1367–1370 (2010).

10. A. E. Price, H.-E. Liang, B. M. Sullivan, R. L. Reinhardt, C. J. Eisley, D. J. Erle, R. M. Locksley, Systemically dispersed innate IL-13-expressing cells in type 2 immunity. *Proc. Natl. Acad. Sci. U.S.A.* **107**, 11489–11494 (2010).

11. M. Ruterbusch, K. B. Pruner, L. Shehata, M. Pepper, In vivo $CD4^+$ T cell differentiation and function: Revisiting the Th1/Th2 paradigm. *Annu. Rev. Immunol.* **38**, 705–725 (2020).

12. P. Zeis, M. Lian, X. Fan, J. S. Herman, D. C. Hernandez, R. Gentek, S. Elias, C. Symowski, K. Knöpper, N. Peltokangas, C. Friedrich, R. Doucet-Ladeveze, A. M. Kabat, R. M. Locksley, D. Voehringer, M. Bajenoff, A. Y. Rudensky, C. Romagnani, D. Grün, G. Gasteiger, In situ maturation and tissue adaptation of type 2 innate lymphoid cell progenitors. *Immunity* **53**, 775–792.e9 (2020).

13. G. Gasteiger, X. Fan, S. Dikiy, S. Y. Lee, A. Y. Rudensky, Tissue residency of innate lymphoid cells in lymphoid and nonlymphoid organs. *Science* **350**, 981–985 (2015).

14. A. B. Molofsky, A. K. Savage, R. M. Locksley, Interleukin-33 in tissue homeostasis, injury, and inflammation. *Immunity* **42**, 1005–1019 (2015).

15. M. F. Molina, M. N. Abdelnabi, T. Fabre, N. H. Shoukry, Type 3 cytokines in liver fibrosis and liver cancer. *Cytokine* **124**, 154497 (2019).

16. T. Fabre, H. Kared, S. L. Friedman, N. H. Shoukry, IL-17A enhances the expression of profibrotic genes through upregulation of the TGF-β receptor on hepatic stellate cells in a JNK-dependent manner. *J. Immunol.* **193**, 3925–3933 (2014).

17. F. Meng, K. Wang, T. Aoyama, S. I. Grivennikov, Y. Paik, D. Scholten, M. Cong, K. Iwaisako, X. Liu, M. Zhang, C. H. Österreicher, F. Stickel, K. Ley, D. A. Brenner, T. Kisseleva, Interleukin-17 signaling in inflammatory, Kupffer cells, and hepatic stellate cells exacerbates liver fibrosis in mice. *Gastroenterology* **143**, 765–776.e3 (2012).

18. D. Tedesco, M. Thapa, C. Y. Chin, Y. Ge, M. Gong, J. Li, S. Gumber, P. Speck, E. J. Elrod, E. M. Burd, W. H. Kitchens, J. F. Magliocca, A. B. Adams, D. S. Weiss, M. Mohamadzadeh, A. Grakoui, Alterations in intestinal microbiota lead to production of interleukin 17 by

intrahepatic γδ T-cell receptor-positive cells and pathogenesis of cholestatic liver disease. *Gastroenterology* **154**, 2178–2193 (2018).

19. A. Wree, M. D. McGeough, M. E. Inzaugarat, A. Eguchi, S. Schuster, C. D. Johnson, C. A. Peña, L. J. Geisler, B. G. Papouchado, H. M. Hoffman, A. E. Feldstein, NLRP3 inflammasome driven liver injury and fibrosis: Roles of IL-17 and TNF in mice. *Hepatology* **67**, 736–749 (2018).

20. D. F. Choy, K. M. Hart, L. A. Borthwick, A. Shikotra, D. R. Nagarkar, S. Siddiqui, G. Jia, C. M. Ohri, E. Doran, K. M. Vannella, C. A. Butler, B. Hargadon, J. C. Sciurba, R. L. Gieseck, R. W. Thompson, S. White, A. R. Abbas, J. Jackman, L. C. Wu, J. G. Egen, L. G. Heaney, T. R. Ramalingam, J. R. Arron, T. A. Wynn, P. Bradding, T_H2 and T_H17 inflammatory pathways are reciprocally regulated in asthma. *Sci. Transl. Med.* **7**, 301ra129 (2015).

21. R. L. Gieseck, T. R. Ramalingam, K. M. Hart, K. M. Vannella, D. A. Cantu, W. Y. Lu, S. Ferreira-González, S. J. Forbes, L. Vallier, T. A. Wynn, Interleukin-13 activates distinct cellular pathways leading to ductular reaction, steatosis, and fibrosis. *Immunity* **45**, 145–158 (2016).

22. T. A. Wynn, K. M. Vannella, Macrophages in tissue repair, regeneration, and fibrosis. *Immunity* **44**, 450–462 (2016).

23. S. Saluzzo, A.-D. Gorki, B. M. J. Rana, R. Martins, S. Scanlon, P. Starkl, K. Lakovits, A. Hladik, A. Korosec, O. Sharif, J. M. Warszawska, H. Jolin, I. Mesteri, A. N. J. McKenzie, S. Knapp, First-breath-induced type 2 pathways shape the lung immune environment. *Cell Rep.* **18**, 1893–1905 (2017).

24. T. Mchedlidze, M. Waldner, S. Zopf, J. Walker, A. L. Rankin, M. Schuchmann, D. Voehringer, A. N. J. McKenzie, M. F. Neurath, S. Pflanz, S. Wirtz, Interleukin-33-dependent innate lymphoid cells mediate hepatic fibrosis. *Immunity* **39**, 357–371 (2013).

25. Z. Tan, Q. Liu, R. Jiang, L. Lv, S. S. Shoto, I. Maillet, V. Quesniaux, J. Tang, W. Zhang, B. Sun, B. Ryffel, Interleukin-33 drives hepatic fibrosis through activation of hepatic stellate cells. *Cell. Mol. Immunol.* **15**, 388–398 (2018).

26. Y. Gao, Y. Liu, M. Yang, X. Guo, M. Zhang, H. Li, J. Li, J. Zhao, IL-33 treatment attenuated diet-induced hepatic steatosis but aggravated hepatic fibrosis. *Oncotarget* **7**, 33649–33661 (2016).

27. J. E. Allen, T. E. Sutherland, Host protective roles of type 2 immunity: Parasite killing and tissue repair, flip sides of the same coin. *Semin. Immunol.* **26**, 329–340 (2014).

28. C. M. Lloyd, R. J. Snelgrove, Type 2 immunity: Expanding our view. *Sci. Immunol.* **3**, eaat1604 (2018).

29. E. Hams, M. E. Armstrong, J. L. Barlow, S. P. Saunders, C. Schwartz, G. Cooke, R. J. Fahy, T. B. Crotty, N. Hirani, R. J. Flynn, D. Voehringer, A. N. J. McKenzie, S. C. Donnelly, P. G. Fallon, IL-25 and type 2 innate lymphoid cells induce pulmonary fibrosis. *Proc. Natl. Acad. Sci. U.S.A.* **111**, 367–372 (2014).

30. M. Forkel, L. Berglin, E. Kekäläinen, A. Carlsson, E. Svedin, J. Michaëlsson, M. Nagasawa, J. S. Erjefält, M. Mori, M. Flodström-Tullberg, A. Bergquist, H.-G. Ljunggren, M. Westgren, U. Lindforss, D. Friberg, C. Jorns, E. Ellis, N. K. Björkström, J. Mjösberg, Composition and functionality of the intrahepatic innate lymphoid cell-compartment in human nonfibrotic and fibrotic livers. *Eur. J. Immunol.* **47**, 1280–1294 (2017).

31. K. M. Cautivo, P. R. Matatia, C. O. Lizama, N. M. Mroz, M. W. Dahlgren, X. Yu, J. Sbierski-Kind, M. T. Taruselli, J. F. Brooks, A. Wade-Vallance, S. E. Caryotakis, A. A. Chang, H.-E. Liang, J. Zikherman, R. M. Locksley, A. B. Molofsky, Interferon gamma constrains type 2 lymphocyte niche boundaries during mixed inflammation. *Immunity* **55**, 254–271.e7 (2022).

32. M. W. Dahlgren, S. W. Jones, K. M. Cautivo, A. Dubinin, J. F. Ortiz-Carpena, S. Farhat, K. S. Yu, K. Lee, C. Wang, A. V. Molofsky, A. D. Tward, M. F. Krummel, T. Peng, A. B. Molofsky, Adventitial stromal cells define group 2 innate lymphoid cell tissue niches. *Immunity* **50**, 707–722.e6 (2019).

33. M. W. Lee, J. I. Odegaard, L. Mukundan, Y. Qiu, A. B. Molofsky, J. C. Nussbaum, K. Yun, R. M. Locksley, A. Chawla, Activated type 2 innate lymphoid cells regulate beige fat biogenesis. *Cell* **160**, 74–87 (2015).

34. J. C. Nussbaum, S. J. Van Dyken, J. von Moltke, L. E. Cheng, A. Mohapatra, A. B. Molofsky, E. E. Thornton, M. F. Krummel, A. Chawla, H.-E. Liang, R. M. Locksley, Type 2 innate lymphoid cells control eosinophil homeostasis. *Nature* **502**, 245–248 (2013).

35. J. Sbierski-Kind, N. Mroz, A. B. Molofsky, Perivascular stromal cells: Directors of tissue immune niches. *Immunol. Rev.* **302**, 10–31 (2021).

36. M. W. Dahlgren, A. B. Molofsky, Adventitial cuffs: Regional hubs for tissue immunity. *Trends Immunol.* **40**, 877–887 (2019).

37. R. Kramann, C. Goettsch, J. Wongboonsin, H. Iwata, R. K. Schneider, C. Kuppe, N. Kaesler, M. Chang-Panesso, F. G. Machado, S. Gratwohl, K. Madhurima, J. D. Hutcheson, S. Jain, E. Aikawa, B. D. Humphreys, Adventitial MSC-like cells are progenitors of vascular smooth muscle cells and drive vascular calcification in chronic kidney disease. *Cell Stem Cell* **19**, 628–642 (2016).

38. T. Tsukui, P. J. Wolters, D. Sheppard, Alveolar fibroblast lineage orchestrates lung inflammation and fibrosis. *Nature* **631**, 627–634 (2024).

39. N. A. Ewing-Crystal, N. M. Mroz, A. Larpthaveesarp, C. O. Lizama, R. Pennington, P. Chiaranunt, J. I. Dennis, A. A. Chang, E. D. Merrill, S. E. Caryotakis, N. Kirthivasan, L. Teo, T. Tsukui, A. Katewa, G. L. McKinsey, S. C. K. Nelson, A. Ciesielska, N. C. Lummis, L. Pintarić, M. W. Dahlgren, A. Atakilit, H. Paidassi, S. Jain, X. Liu, D. Xu, M. K. Aghi, J. A. Bourne, J. T. Paz, R. Daneman, F. F. Gonzalez, D. Sheppard, A. V. Molofsky, T. D. Arnold, A. B. Molofsky, Dynamic fibroblast–immune interactions shape recovery after brain injury. *Nature* **646**, 934–944 (2025).

40. M. W. Dahlgren, A. B. Molofsky, All along the watchtower: Group 2 innate lymphoid cells in allergic responses. *Curr. Opin. Immunol.* **54**, 13–19 (2018).

41. R. Dobie, J. R. Wilson-Kanamori, B. E. P. Henderson, J. R. Smith, K. P. Matchett, J. R. Portman, K. Wallenborg, S. Picelli, A. Zagorska, S. V. Pendem, T. E. Hudson, M. M. Wu, G. R. Budas, D. G. Breckenridge, E. M. Harrison, D. J. Mole, S. J. Wigmore, P. Ramachandran, C. P. Ponting, S. A. Teichmann, J. C. Marioni, N. C. Henderson, Single-cell transcriptomics

uncovers zonation of function in the mesenchyme during liver fibrosis. *Cell Rep.* **29**, 1832–1847.e8 (2019).

42. T. Tsuchida, Y. A. Lee, N. Fujiwara, M. Ybanez, B. Allen, S. Martins, M. I. Fiel, N. Goossens, H.-I. Chou, Y. Hoshida, S. L. Friedman, A simple diet- and chemical-induced murine NASH model with rapid progression of steatohepatitis, fibrosis and liver cancer. *J. Hepatol.* **69**, 385–395 (2018).

43. A. Ghallab, M. Myllys, C. H. Holland, A. Zaza, W. Murad, R. Hassan, Y. A. Ahmed, T. Abbas, E. A. Abdelrahim, K. M. Schneider, M. Matz-Soja, J. Reinders, R. Gebhardt, M.-L. Berres, M. Hatting, D. Drasdo, J. Saez-Rodriguez, C. Trautwein, J. G. Hengstler, Influence of liver fibrosis on lobular zonation. *Cells* **8**, 1556 (2019).

44. J. M. Saito, J. J. Maher, Bile duct ligation in rats induces biliary expression of cytokine-induced neutrophil chemoattractant. *Gastroenterology* **118**, 1157–1168 (2000).

45. S. E. Di Carlo, L. Peduto, The perivascular origin of pathological fibroblasts. *J. Clin. Investig.* **128**, 54–63 (2018).

46. A. T. Krishnamurty, J. A. Shyer, M. Thai, V. Gandham, M. B. Buechler, Y. A. Yang, R. N. Pradhan, A. W. Wang, P. L. Sanchez, Y. Qu, B. Breart, C. Chalouni, D. Dunlap, J. Ziai, J. Elstrott, N. Zacharias, W. Mao, R. K. Rowntree, J. Sadowsky, G. D. Lewis, T. H. Pillow, B. Y. Nabet, R. Banchereau, L. Tam, R. Caothien, N. Bacarro, M. Roose-Girma, Z. Modrusan, S. Mariathasan, S. Müller, S. J. Turley, LRRC15^{+} myofibroblasts dictate the stromal setpoint to suppress tumour immunity. *Nature* **611**, 148–154 (2022).

47. T. Tsukui, K. H. Sun, J. B. Wetter, J. R. Wilson-Kanamori, L. A. Hazelwood, N. C. Henderson, T. S. Adams, J. C. Schupp, S. D. Poli, I. O. Rosas, N. Kaminski, M. A. Matthay, P. J. Wolters, D. Sheppard, Collagen-producing lung cell atlas identifies multiple subsets with distinct localization and relevance to fibrosis. *Nat. Commun.* **11**, 1920 (2020).

48. P. K. Langston, Y. Sun, B. A. Ryback, A. L. Mueller, B. M. Spiegelman, C. Benoist, D. Mathis, Regulatory T cells shield muscle mitochondria from interferon-γ–mediated damage to promote the beneficial effects of exercise. *Sci. Immunol.* **8**, eadi5377 (2023).

49. A. L. Degryse, H. Tanjore, X. C. Xu, V. V. Polosukhin, B. R. Jones, F. B. McMahon, L. A. Gleaves, T. S. Blackwell, W. E. Lawson, Repetitive intratracheal bleomycin models several features of idiopathic pulmonary fibrosis. *Am. J. Physiol. Lung Cell. Mol. Physiol.* **299**, L442–L452 (2010).

50. M. Lochner, L. Peduto, M. Cherrier, S. Sawa, F. Langa, R. Varona, D. Riethmacher, M. Si-Tahar, J. P. Di Santo, G. Eberl, In vivo equilibrium of proinflammatory IL-17^{+} and regulatory IL-10^{+} Foxp3^{+} RORγt^{+} T cells. *J. Exp. Med.* **205**, 1381–1393 (2008).

51. K. Hirota, J. H. Duarte, M. Veldhoen, E. Hornsby, Y. Li, D. J. Cua, H. Ahlfors, C. Wilhelm, M. Tolaini, U. Menzel, A. Garefalaki, A. J. Potocnik, B. Stockinger, Fate mapping of IL-17-producing T cells in inflammatory responses. *Nat. Immunol.* **12**, 255–263 (2011).

52. K. M. Hart, T. Fabre, J. C. Sciurba, R. L. Gieseck, L. A. Borthwick, K. M. Vannella, T. H. Acciani, R. De Queiroz Prado, R. W. Thompson, S. White, G. Soucy, M. Bilodeau, T. R. Ramalingam, J. R. Arron, N. H. Shoukry, T. A. Wynn, Type 2 immunity is protective in metabolic disease but exacerbates NAFLD collaboratively with TGF-β. *Sci. Transl. Med.* **9**, eaal3694 (2017).

53. S. J. Van Dyken, A. Mohapatra, J. C. Nussbaum, A. B. Molofsky, E. E. Thornton, S. F. Ziegler, A. N. J. McKenzie, M. F. Krummel, H.-E. Liang, R. M. Locksley, Chitin activates parallel immune modules that direct distinct inflammatory responses via innate lymphoid type 2 and γδ T cells. *Immunity* **40**, 414–424 (2014).

54. J. J. Barron, N. M. Mroz, S. E. Taloma, M. W. Dahlgren, J. F. Ortiz-Carpena, M. G. Keefe, C. C. Escoubas, L. C. Dorman, I. D. Vainchtein, P. Chiaranunt, M. E. Kotas, T. J. Nowakowski, K. J. Bender, A. B. Molofsky, A. V. Molofsky, Group 2 innate lymphoid cells promote inhibitory synapse development and social behavior. *Science* **386**, eadi1025 (2025).

55. P. Ramachandran, R. Dobie, J. R. Wilson-Kanamori, E. F. Dora, B. E. P. Henderson, N. T. Luu, J. R. Portman, K. P. Matchett, M. Brice, J. A. Marwick, R. S. Taylor, M. Efremova, R. Vento-Tormo, N. O. Carragher, T. J. Kendall, J. A. Fallowfield, E. M. Harrison, D. J. Mole, S. J. Wigmore, P. N. Newsome, C. J. Weston, J. P. Iredale, F. Tacke, J. W. Pollard, C. P. Ponting,

J. C. Marioni, S. A. Teichmann, N. C. Henderson, Resolving the fibrotic niche of human liver cirrhosis at single-cell level. *Nature* **575**, 512–518 (2019).

56. A. B. Molofsky, J. C. Nussbaum, H.-E. Liang, S. J. Van Dyken, L. E. Cheng, A. Mohapatra, A. Chawla, R. M. Locksley, Innate lymphoid type 2 cells sustain visceral adipose tissue eosinophils and alternatively activated macrophages. *J. Exp. Med.* **210**, 535–549 (2013).

57. M. Mohrs, B. Ledermann, G. Köhler, A. Dorfmüller, A. Gessner, F. Brombacher, Differences between IL-4- and IL-4 receptor α-deficient mice in chronic leishmaniasis reveal a protective role for IL-13 receptor signaling. *J. Immunol.* **162**, 7302–7308 (1999).

58. T. Fabre, A. M. S. Barron, S. M. Christensen, S. Asano, K. Bound, M. P. Lech, M. H. Wadsworth, X. Chen, C. Wang, J. Wang, J. McMahon, F. Schlerman, A. White, K. M. Kravarik, A. J. Fisher, L. A. Borthwick, K. M. Hart, N. C. Henderson, T. A. Wynn, K. Dower, Identification of a broadly fibrogenic macrophage subset induced by type 3 inflammation. *Sci. Immunol.* **8**, eadd8945 (2023).

59. F. F. De Ponti, A. Bujko, Z. Liu, P. J. Collins, S. Schuermans, C. Maueroder, S. Amstelveen, T. Thoné, L. Martens, J. G. McKendrick, P. A. Louwe, A. Sànchez Cruz, W. Saelens, K. P. Matchett, K. J. Waller, C. Zwicker, A. Buglar-Lamb, B. Vanneste, F. Parmentier, M. B. A. Latib, A. Remmerie, L. Kertesz, A. Kremer, J. Verbeke, D. H. Ipsen, D. R. Pfister, Z. Liu, M. Guilliams, N. C. Henderson, K. Ravichandran, P. E. Marques, C. L. Scott, Spatially restricted and ontogenically distinct hepatic macrophages are required for tissue repair. *Immunity* **58**, 362–380.e10 (2025).

60. C. Xu, S. Li, T. S. Fulford, S. N. Christo, L. K. Mackay, D. H. D. Gray, A. P. Uldrich, D. G. Pellicci, D. I. Godfrey, H.-F. Koay, Expansion of MAIT cells in the combined absence of NKT and γδ-T cells. *Mucosal Immunol.* **16**, 446–461 (2023).

61. I. I. Ivanov, B. S. McKenzie, L. Zhou, C. E. Tadokoro, A. Lepelley, J. J. Lafaille, D. J. Cua, D. R. Littman, The orphan nuclear receptor RORγt directs the differentiation program of proinflammatory IL-17^{+} T helper cells. *Cell* **126**, 1121–1133 (2006).

62. D. R. Withers, M. R. Hepworth, X. Wang, E. C. Mackley, E. E. Halford, E. E. Dutton, C. L. Marriott, V. Brucklacher-Waldert, M. Veldhoen, J. Kelsen, R. N. Baldassano, G. F. Sonnenberg, Transient inhibition of ROR-γt therapeutically limits intestinal inflammation by reducing TH17 cells and preserving group 3 innate lymphoid cells. *Nat. Med.* **22**, 319–323 (2016).

63. C. R. Merritt, G. T. Ong, S. E. Church, K. Barker, P. Danaher, G. Geiss, M. Hoang, J. Jung, Y. Liang, J. McKay-Fleisch, K. Nguyen, Z. Norgaard, K. Sorg, I. Sprague, C. Warren, S. Warren, P. J. Webster, Z. Zhou, D. R. Zollinger, D. L. Dunaway, G. B. Mills, J. M. Beechem, Multiplex digital spatial profiling of proteins and RNA in fixed tissue. *Nat. Biotechnol.* **38**, 586–599 (2020).

64. P. Danaher, Y. Kim, B. Nelson, M. Griswold, Z. Yang, E. Piazza, J. M. Beechem, Advances in mixed cell deconvolution enable quantification of cell types in spatial transcriptomic data. *Nat. Commun.* **13**, 385 (2022).

65. M. B. Buechler, R. N. Pradhan, A. T. Krishnamurty, C. Cox, A. K. Calviello, A. W. Wang, Y. A. Yang, L. Tam, R. Caothien, M. Roose-Girma, Z. Modrusan, J. R. Arron, R. Bourgon, S. Müller, S. J. Turley, Cross-tissue organization of the fibroblast lineage. *Nature* **593**, 575–579 (2021).

66. J. Paris, N. C. Henderson, Liver zonation, revisited. *Hepatology* **76**, 1219–1230 (2022).

67. K. M. Cautivo, C. A. Steer, A. B. Molofsky, Immune outposts in the adventitia: One foot in sea and one on shore. *Curr. Opin. Immunol.* **64**, 34–41 (2020).

68. J. Rustenhoven, A. Drieu, T. Mamuladze, K. A. de Lima, T. Dykstra, M. Wall, Z. Papadopoulos, M. Kanamori, A. F. Salvador, W. Baker, M. Lemieux, S. D. Mesquita, A. Cugurra, J. Fitzpatrick, S. Sviben, R. Kossina, P. Bayguinov, R. R. Townsend, Q. Zhang, P. Erdmann-Gilmore, I. Smirnov, M.-B. Lopes, J. Herz, J. Kipnis, Functional characterization of the dural sinuses as a neuroimmune interface. *Cell* **184**, 1000–1016.e27 (2021).

69. B. S. Kim, M. C. Siracusa, S. A. Saenz, M. Noti, L. A. Monticelli, G. F. Sonnenberg, M. R. Hepworth, A. S. Van Voorhees, M. R. Comeau, D. Artis, TSLP elicits IL-33-independent

innate lymphoid cell responses to promote skin inflammation. *Sci. Transl. Med.* **5**, 170ra16 (2013).

70. K. M. Vannella, T. R. Ramalingam, L. A. Borthwick, L. Barron, K. M. Hart, R. W. Thompson, K. N. Kindrachuk, A. W. Cheever, S. White, A. L. Budelsky, M. R. Comeau, D. E. Smith, T. A. Wynn, Combinatorial targeting of TSLP, IL-25, and IL-33 in type 2 cytokine-driven inflammation and fibrosis. *Sci. Transl. Med.* **8**, 337ra65 (2016).

71. S. J. Van Dyken, R. M. Locksley, Interleukin-4- and interleukin-13-mediated alternatively activated macrophages: Roles in homeostasis and disease. *Annu. Rev. Immunol.* **31**, 317–343 (2013).

72. D. Karo-Atar, A. Bordowitz, O. Wand, M. Pasmanik-Chor, I. E. Fernandez, M. Itan, R. Frenkel, D. R. Herbert, F. D. Finkelman, O. Eickelberg, A. Munitz, A protective role for IL-13 receptor α 1 in bleomycin-induced pulmonary injury and repair. *Mucosal Immunol.* **9**, 240–253 (2016).

73. M. S. Wilson, S. K. Madala, T. R. Ramalingam, B. R. Gochuico, I. O. Rosas, A. W. Cheever, T. A. Wynn, Bleomycin and IL-1β–mediated pulmonary fibrosis is IL-17A dependent. *J. Exp. Med.* **207**, 535–552 (2010).

74. T. Fabre, M. F. Molina, G. Soucy, J. P. Goulet, B. Willems, J. P. Villeneuve, M. Bilodeau, N. H. Shoukry, Type 3 cytokines IL-17A and IL-22 drive TGF-β–dependent liver fibrosis. *Sci. Immunol.* **3**, eaar7754 (2018).

75. Z. Tan, X. Qian, R. Jiang, Q. Liu, Y. Wang, C. Chen, X. Wang, B. Ryffel, B. Sun, IL-17A plays a critical role in the pathogenesis of liver fibrosis through hepatic stellate cell activation. *J. Immunol.* **191**, 1835–1844 (2013).

76. S. Marinović, M. Lenartić, K. Mladenić, M. Šestan, I. Kavazović, A. Benić, M. Krapić, L. Rindlisbacher, M. Cokarić Brdovčak, C. Sparano, G. Litscher, T. T. Wensveen, I. Mikolašević, D. Fučkar Čupić, L. Bilić-Zulle, A. Steinle, A. Waisman, A. Hayday, S. Tugues, B. Becher, B. Polić, F. M. Wensveen, NKG2D-mediated detection of metabolically stressed

hepatocytes by innate-like T cells is essential for initiation of NASH and fibrosis. *Sci. Immunol.* **8**, eadd1599 (2023).

77. J. Magdaleno-Tapial, C. López-Martí, J. M. Ortiz-Salvador, P. Hernández-Bel, J. J. Tamarit-García, M. Diago-Madrid, J. L. Sánchez-Carazo, A. Pérez-Ferriols, Can secukinumab improve liver fibrosis? A pilot prospective study of 10 psoriatic patients. *Dermatol. Ther.* **34**, e15065 (2021).

78. M. Guilliams, J. Bonnardel, B. Haest, B. Vanderborght, C. Wagner, A. Remmerie, A. Bujko, L. Martens, T. Thoné, R. Browaeys, F. F. De Ponti, B. Vanneste, C. Zwicker, F. R. Svedberg, T. Vanhalewyn, A. Gonçalves, S. Lippens, B. Devriendt, E. Cox, G. Ferrero, V. Wittamer, A. Willaert, S. J. F. Kaptein, J. Neyts, K. Dallmeier, P. Geldhof, S. Casaert, B. Deplancke, P. ten Dijke, A. Hoorens, A. Vanlander, F. Berrevoet, Y. Van Nieuwenhove, Y. Saeys, W. Saelens, H. Van Vlierberghe, L. Devisscher, C. L. Scott, Spatial proteogenomics reveals distinct and evolutionarily conserved hepatic macrophage niches. *Cell* **185**, 379–396.e38 (2022).

79. A. Remmerie, L. Martens, T. Thoné, A. Castoldi, R. Seurinck, B. Pavie, J. Roels, B. Vanneste, S. De Prijck, M. Vanhockerhout, M. B. A. Latib, L. Devisscher, A. Hoorens, J. Bonnardel, N. Vandamme, A. Kremer, P. Borghgraef, H. Van Vlierberghe, S. Lippens, E. Pearce, Y. Saeys, C. L. Scott, Osteopontin expression identifies a subset of recruited macrophages distinct from Kupffer cells in the fatty liver. *Immunity* **53**, 641–657.e14 (2020).

80. A. Gola, M. G. Dorrington, E. Speranza, C. Sala, R. M. Shih, A. J. Radtke, H. S. Wong, A. P. Baptista, J. M. Hernandez, G. Castellani, I. D. C. Fraser, R. N. Germain, Commensal-driven immune zonation of the liver promotes host defence. *Nature* **589**, 131–136 (2021).

81. L. D. Faustino, J. W. Griffith, R. A. Rahimi, K. Nepal, D. L. Hamilos, J. L. Cho, B. D. Medoff, J. J. Moon, D. A. A. Vignali, A. D. Luster, Interleukin-33 activates regulatory T cells to suppress innate γδ T cell responses in the lung. *Nat. Immunol.* **21**, 1371–1383 (2020).

82. S. Hemmers, M. Schizas, A. Y. Rudensky, T reg cell-intrinsic requirements for ST2 signaling in health and neuroinflammation. *J. Exp. Med.* **218**, e20201234 (2021).

83. R. R. Ricardo-Gonzalez, S. J. Van Dyken, C. Schneider, J. Lee, J. C. Nussbaum, H.-E. Liang, D. Vaka, W. L. Eckalbar, A. B. Molofsky, D. J. Erle, R. M. Locksley, Tissue signals imprint ILC2 identity with anticipatory function. *Nat. Immunol.* **19**, 1093–1099 (2018).

84. I. C. Boothby, M. J. Kinet, D. P. Boda, E. Y. Kwan, S. Clancy, J. N. Cohen, I. Habrylo, M. M. Lowe, M. Pauli, A. E. Yates, J. D. Chan, H. W. Harris, I. M. Neuhaus, T. H. McCalmont, A. B. Molofsky, M. D. Rosenblum, Early-life inflammation primes a T helper 2 cell–fibroblast niche in skin. *Nature* **599**, 667–672 (2021).

85. Y. P. S. Goh, N. C. Henderson, J. E. Heredia, A. R. Eagle, J. I. Odegaard, N. Lehwald, K. D. Nguyen, D. Sheppard, L. Mukundan, R. M. Locksley, A. Chawla, Eosinophils secrete IL-4 to facilitate liver regeneration. *Proc. Natl. Acad. Sci. U.S.A.* **110**, 9914–9919 (2013).

86. L. Xu, Y. Yang, Y. Wen, J. M. Jeong, C. Emontzpohl, C. L. Atkins, Z. Sun, K. L. Poulsen, D. R. Hall, J. Steve Bynon, B. Gao, W. M. Lee, J. Rule, E. A. Jacobsen, H. Wang, C. Ju, Hepatic recruitment of eosinophils and their protective function during acute liver injury. *J. Hepatol.* **77**, 344–352 (2022).

87. Y. Yang, L. Xu, C. Atkins, L. Kuhlman, J. Zhao, J. M. Jeong, Y. Wen, N. Moreno, K. H. Kim, Y. A. An, F. Wang, S. Bynon, V. Villani, B. Gao, F. Brombacher, R. Harris, H. K. Eltzschig, E. Jacobsen, C. Ju, Novel IL-4/HB-EGF-dependent crosstalk between eosinophils and macrophages controls liver regeneration after ischaemia and reperfusion injury. *Gut* **73**, 1543–1553 (2024).

88. M. Rau, A.-K. Schilling, J. Meertens, I. Hering, J. Weiss, C. Jurowich, T. Kudlich, H. M. Hermanns, H. Bantel, N. Beyersdorf, A. Geier, Progression from nonalcoholic fatty liver to nonalcoholic steatohepatitis is marked by a higher frequency of Th17 cells in the liver and an increased Th17/resting regulatory T cell ratio in peripheral blood and in the liver. *J. Immunol.* **196**, 97–105 (2016).

89. L. Madisen, T. A. Zwingman, S. M. Sunkin, S. W. Oh, H. A. Zariwala, H. Gu, L. L. Ng, R. D. Palmiter, M. J. Hawrylycz, A. R. Jones, E. S. Lein, H. Zeng, A robust and high-throughput Cre reporting and characterization system for the whole mouse brain. *Nat. Neurosci.* **13**, 133–140 (2010).

90. T. Buch, F. L. Heppner, C. Tertilt, T. J. A. J. Heinen, M. Kremer, F. T. Wunderlich, S. Jung, A. Waisman, A Cre-inducible diphtheria toxin receptor mediates cell lineage ablation after toxin administration. *Nat. Methods* **2**, 419–426 (2005).

91. I. D. Vainchtein, G. Chin, F. S. Cho, K. W. Kelley, J. G. Miller, E. C. Chien, S. A. Liddelow, P. T. Nguyen, H. Nakao-Inoue, L. C. Dorman, O. Akil, S. Joshita, B. A. Barres, J. T. Paz, A. B. Molofsky, A. V. Molofsky, Astrocyte-derived interleukin-33 promotes microglial synapse engulfment and neural circuit development. *Science* **359**, 1269–1273 (2018).

92. T. A. Reese, H.-E. Liang, A. M. Tager, A. D. Luster, N. Van Rooijen, D. Voehringer, R. M. Locksley, Chitin induces accumulation in tissue of innate immune cells associated with allergy. *Nature* **447**, 92–96 (2007).

93. H.-E. Liang, R. L. Reinhardt, J. K. Bando, B. M. Sullivan, I.-C. Ho, R. M. Locksley, Divergent expression patterns of IL-4 and IL-13 define unique functions in allergic immunity. *Nat. Immunol.* **13**, 58–66 (2012).

94. C. Schneider, J. Lee, S. Koga, R. R. Ricardo-Gonzalez, J. C. Nussbaum, L. K. Smith, S. A. Villeda, H. E. Liang, R. M. Locksley, Tissue-resident group 2 innate lymphoid cells differentiate by layered ontogeny and in situ perinatal priming. *Immunity* **50**, 1425–1438.e5 (2019).

95. L. A. Kalekar, J. N. Cohen, N. Prevel, P. M. Sandoval, A. N. Mathur, J. M. Moreau, M. M. Lowe, A. Nosbaum, P. J. Wolters, A. Haemel, F. Boin, M. D. Rosenblum, Regulatory T cells in skin are uniquely poised to suppress profibrotic immune responses. *Sci. Immunol.* **4**, eaaw2910 (2019).

96. J. Zhu, D. Jankovic, A. J. Oler, G. Wei, S. Sharma, G. Hu, L. Guo, R. Yagi, H. Yamane, G. Punkosdy, L. Feigenbaum, K. Zhao, W. E. Paul, The transcription factor T-bet is induced by multiple pathways and prevents an endogenous Th2 cell program during Th1 cell responses. *Immunity* **37**, 660–673 (2012).

97. M. Cassandras, C. Wang, J. Kathiriya, T. Tsukui, P. Matatia, M. Matthay, P. Wolters, A. Molofsky, D. Sheppard, H. Chapman, T. Peng, $Gli1^{+}$ mesenchymal stromal cells form a

pathological niche to promote airway progenitor metaplasia in the fibrotic lung. *Nat. Cell Biol.* **22**, 1295–1306 (2020).

98. T. Suda, D. Liu, Hydrodynamic gene delivery: Its principles and applications. *Mol. Ther.* **15**, 2063–2069 (2007).

99. P. Aboyoun, N. Ortogero, M. Griswold, Z. Yang, NanoStringNCTools: NanoString nCounter Tools (2024); https://bioconductor.org/packages/release/bioc/html/NanoStringNCTools.html.

100. K. Blighe, S. Rana, M. Lewis, EnhancedVolcano: Publication-ready volcano plots with enhanced colouring and labeling (2018); https://github.com/kevinblighe/EnhancedVolcano.

101. M. Griswold, P. Danaher, SpatialDecon: Deconvolution of mixed cells from spatial and/or bulk gene expression data (2024); https://rdrr.io/bioc/SpatialDecon/.

102. H. Yoshida, C. A. Lareau, R. N. Ramirez, S. A. Rose, B. Maier, A. Wroblewska, F. Desland, A. Chudnovskiy, A. Mortha, C. Dominguez, J. Tellier, E. Kim, D. Dwyer, S. Shinton, T. Nabekura, Y. L. Qi, B. Yu, M. Robinette, K. W. Kim, A. Wagers, A. Rhoads, S. L. Nutt, B. D. Brown, S. Mostafavi, J. D. Buenrostro, C. Benoist, The cis-regulatory atlas of the mouse immune system. *Cell* **176**, 897–912.e20 (2019).

103. I. Mederacke, D. H. Dapito, S. Affò, H. Uchinami, R. F. Schwabe, High-yield and high-purity isolation of hepatic stellate cells from normal and fibrotic mouse livers. *Nat. Protoc.* **10**, 305–315 (2015).
